# Effective Cellular Transport of *Ortho*-Halogenated Sulfonamide Derivatives of Metformin Is Related to Improved Antiproliferative Activity and Apoptosis Induction in MCF-7 Cells

**DOI:** 10.3390/ijms21072389

**Published:** 2020-03-30

**Authors:** Magdalena Markowicz-Piasecka, Ibrahim Komeil, Johanna Huttunen, Joanna Sikora, Kristiina M. Huttunen

**Affiliations:** 1 Laboratory of Bioanalysis, Department of Pharmaceutical Chemistry, Drug Analysis and Radiopharmacy, Medical University of Lodz, ul. Muszyńskiego1, 90-151 Lodz, Poland; joanna.sikora@umed.lodz.pl; 2 Department of Pharmaceutics, Faculty of Pharmacy and Drug Manufacturing, Pharos University, Alexandria 21311, Egypt; ibrahim.komeil@pua.edu.eg; 3 School of Pharmacy, Faculty of Health Sciences, University of Eastern Finland, Yliopistonranta 1C, POB 1627, 70211 Kuopio, Finland; johanna.huttunen@uef.fi (J.H.); kristiina.huttunen@uef.fi (K.M.H.)

**Keywords:** biguanides, metformin, organic cation transporters (OCT), cellular uptake, cytotoxicity, apoptosis

## Abstract

Metformin is a substrate for plasma membrane monoamine transporters (PMAT) and organic cation transporters (OCTs); therefore, the expression of these transporters and interactions between them may affect the uptake of metformin into tumor cells and its anticancer efficacy. The aim of this study was to evaluate how chemical modification of metformin scaffold into benzene sulfonamides with halogen substituents (compounds **1**–**9**) may affect affinity towards OCTs, cellular uptake in two breast cancer cell lines (MCF-7 and MDA-MB-231) and antiproliferative efficacy of metformin. The uptake of most sulfonamides was more efficient in MCF-7 cells than in MDA-MB-231 cells. The presence of a chlorine atom in the aromatic ring contributed to the highest uptake in MCF-7 cells. For instance, the uptake of compound **1** with *o*-chloro substituent in MCF-7 cells was 1.79 ± 0.79 nmol/min/mg protein, while in MDA-MB-231 cells, the uptake was considerably lower (0.005 ± 0.0005 nmol/min/mg protein). The elevated uptake of tested compounds in MCF-7 was accompanied by high antiproliferative activity, with compound **1** being the most active (IC_50_ = 12.6 ± 1.2 µmol/L). Further studies showed that inhibition of MCF-7 growth is associated with the induction of early and late apoptosis and cell cycle arrest at the G0/G1 phase. In summary, the chemical modification of the biguanide backbone into halogenated sulfonamides leads to improved transporter-mediated cellular uptake in MCF-7 and contributes to the greater antiproliferative potency of studied compounds through apoptosis induction and cell cycle arrest.

## 1. Introduction

Metformin represents a first-line strategy for the treatment of type 2 diabetes (T2DM), mainly because of high clinical value regarding glycemic control, safety profile and low costs [1]. The basic metformin activity, glucose-lowering properties, stem from several mechanisms including: inhibition of hepatic gluconeogenesis via an LKB1/AMP-activated protein kinase–mediated mechanism, an increase in tissues’ glucose consumption and sensitivity of insulin and decrease in intestinal glucose absorption. In addition, metformin is characterized by multidirectional biological activity, which brings beneficial effects on mortality rates in diabetic patients, improves the serum lipids profile and functions of the endothelium, as well as stimulates gene expression responsible for cellular antioxidant defense mechanisms [2]. Therefore, its role as a therapeutic agent has been expanding to the treatment of pre-diabetes, gestational diabetes and polycystic ovary syndrome, as well as the treatment or prevention of preeclampsia [1].

One issue that might also be of great importance is the fact that metformin may exert cancer chemopreventive activity through inhibition of transformative and hyper-proliferative processes, initiating carcinogenesis [3]. A review of an exponentially growing number of publications allows to conclude that anticancer effects of metformin stem from several mechanisms: activation of LKB1/AMPK pathway and subsequent inhibition of the mammalian target of rapamycin (mTOR), induction of cell cycle arrest or apoptosis, inhibition of protein synthesis, activation of the immune system and a possible eradication of cancer stem cells [4]. The data of in vitro and in vivo studies suggest that metformin may suppress cancer cell growth and reduce the risk of some types of cancers, including breast, colon, pancreas and liver cancers, and might improve cancer prognosis [4]. A large number of publications are those that concern the inhibitory effect of metformin on the growth of breast cancer cells [5,6,7]. In addition, metformin may reduce the mortality from cancers, increase the response to treatment in cancer cells when using radiotherapy and chemotherapy and reduce the malignancy and the likelihood of relapse [8].

The structure and hydrophilic character of metformin determine its pharmacokinetic properties. Since metformin exists in a positively charged form under physiological conditions, passive diffusion of the drug through biological membranes is hindered. Transport of metformin involves an uptake process via transporters [9]. For instance, the intestinal absorption of metformin is primarily mediated by plasma membrane monoamine transporter (PMAT), while the hepatic uptake of metformin occurs primarily with organic cation transporter 1 (OCT1). Metformin is excreted mainly through the kidneys, and the uptake of metformin from the circulation into renal epithelial cells is facilitated by OCT2. Apart from OCTs and PMAT transporters, metformin is carried into the cells also by other carriers, such as multidrug and toxin extrusion 1 and 2 (MATE1 and 2), depending on the tissue [10]. As mentioned before, metformin was found to possess antiproliferative potential in some cell lines [11]; therefore, scientists started to search for biguanide derivatives with improved antiproliferative properties. For instance, Cheng et al. [12] synthesized novel alkylated derivatives of metformin containing a triphenylphosphonium cation (TPP^+^) and showed that these compounds were more effective than metformin in inhibition of proliferation of pancreatic ductal adenocarcinoma. However, based on the number of transporters engaged in cellular uptake of metformin into various tissues, we can expect that the presence of transporters and interactions between them may affect the uptake of metformin into tumor cells and, as a consequence, affect its anticancer efficacy [13]. This hypothesis has been confirmed by Cai et al. [14], who have demonstrated that expression of OCT transporters correlates with the antiproliferative and antitumor efficacy of metformin in breast cancer. Additionally, one of our previous papers [13] showed that a new drug design strategy incorporating an alkyl sulfenamide chain into the metformin backbone contributed to greater affinity towards OCTs, better cellular uptake and antiproliferative properties of the parent drug.

Several epidemiologic studies have provided correlations between metformin administration and a reduction in breast cancer incidence in prediabetic and T2DM patients [15,16]. However, it is little known which patient populations and types of tumors are susceptible to metformin treatment. Taking into consideration that metformin cellular uptake is based on several types of transporters [14], it is also important to evaluate the efficacy of intratumoral uptake of the drug. However, most preclinical in vivo studies have paid little attention to the effectiveness of metformin accumulation in tumors. One of the few exceptions is a study of Checkley et al. [17], who provided evidence that OCT2 expression in tumor tissue may predict metformin uptake and tumor response.

**Figure 1:**
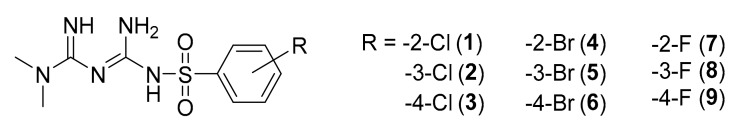
Chemical structure of tested biguanide derivatives—metformin sulfonyl halides (**1**–**9**).

In this paper, we verify the hypothesis whether chemical modification of metformin scaffold into sulfonamides may improve cellular uptake and inhibit breast cancer cell growth. Nine sulfonamide metformin derivatives differing with a halogen substituent in the benzene ring (compounds **1**–**9**, Figure 1) were examined, and their affinity towards organic cation transporters (OCTs) was determined. In addition, the uptake of these compounds in two human breast cancer cell lines, MCF-7 and MDA-MB-231, was comprehensively evaluated. Hence, this is the first study reporting how the chemical modification of biguanide scaffold into sulfonamides can increase transporter-mediated cellular uptake of metformin and affect antiproliferative potency of the studied compounds.

## 2. Results and Discussion

### 2.1. Expression of MATE1–2, PMAT and the Function of OCTs in MCF-7 and MDA-MB-231 Cells

The expression of PMAT and MATE in MCF-7 and MDA-MB-231 cells at the RNA level was determined using RT-PCR (Supplementary Figure S1). Our previous studies [13] revealed that, among OCT transporters, OCT3 was predominant in MDA-MB-231 cells, while, in the case of MCF-7, we were not able to detect any of the OCT transporters. In the case of MATE transporters, we found similar significant expressions of MATE1 in both cell lines. Similar to the other study [14], a negligible expression of MATE2 transporters was reported. With regards to the expression of PMAT transporters, its mean normalized expression was approximately three-fold higher in MCF-7 cells than in MDA-MB-231 cells (Supplementary Figure S1). Collectively, these data suggest that there is a substantial variability in expression of metformin transporters among several breast cancer cell lines, which consequently, may contribute to changes in cellular uptake of the drug and limit the antiproliferative effects of metformin.

The function of OCTs in MCF-7 and MDA-MB-231 cells was evaluated previously [13] using a radiolabeled [^14^C] choline. Time-dependent and concentration-dependent characterizations of [^14^C] choline uptakes were also conducted [13].

### 2.2. Inhibition of [^14^C]Choline Uptake in MCF-7 and MDA-MB-231 Cells

The affinity of compounds **1**–**9** to bind to OCT transporters was determined using a competitive inhibition assay with OCT substrate [^14^C] choline. The potential of studied compounds to bind to OCTs is presented in Table 1 and expressed as the half-maximal inhibitory concentration (IC_50_). Compound **1** with *o*-chloro substituent in the aromatic ring presented the highest affinity for OCTs in both MCF-7 and MDA-MB-231 cells among chloro-benzenesulfonamides (IC_50_ values 1053 ± 14.2 μmol/L and 2135 ± 14.0 μmol/L, respectively).

The affinity of compound **1** towards OCTs is much higher than that of the parent drug, metformin, for which the maximal inhibition of [^14^C] choline uptake was 34.3% for MCF-7 cells and 29.9% for MDA-MB-231 cells at a concentration of 2400 μmol/L. Compound **5** with *m*-bromo substituent in the aromatic ring was found to possess the highest affinity towards OCTs in both cell lines, expressed as the lowest IC_50_ values (888.5 ± 11.4 μmol/L and 593.1 ± 15.0 μmol/L). The other chloro- and bromo-derivatives were characterized by moderate OCTs’ binding properties. In the case of fluoro-benzene sulfonamides (compounds **7**–**9**), compounds with a fluorine atom in the *para* position presented the highest affinity towards OCTs in MCF-7 and MDA-MB-231 cells (IC_50_ values 1057 ± 7.5 μmol/L and 1383 ± 14.0 μmol/L, respectively). Of the tested sulfonamides, compounds **7** and **8** showed the lowest affinity towards OCTs in MCF-7 cells.

### 2.3. Cellular Uptake of Metformin Derivatives

#### 2.3.1. General Characterization

**Figure 2:**
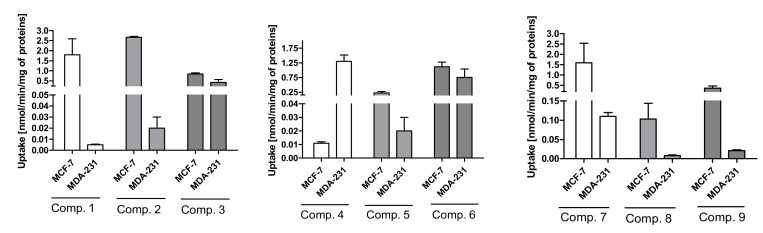
The uptake of sulfonamide derivatives of metformin into MCF-7 and MDA-MB-231 cells at an 800 μmol/L concentration after 10 min incubation at 37 °C. Metformin uptake was 0.107 ± 0.006 nmol/min/mg of proteins in MCF-7 cells and 0.117 ± 0.010 nmol/min/mg of proteins in MDA-MB-231 cells [13].

The first step of the studies included an establishment of a relationship between the concentration of the tested compound and its cellular uptake. These studies enable us to assess whether metformin derivatives are transported into MCF-7 and MDA-MB-231 cells or only bound to them on the cell surface. Figure 2 presents the uptake of sulfonamides **1**–**9** at a concentration of 800 μmol/L after 10-minute incubation. As seen in Figure 2, all chloro-substituted benzenesulfonamides (compounds **1**–**3**) were uptaken efficiently in MCF-7 cells. For instance, the uptake of compound **2** was 2.669 ± 0.040 nmol/min/mg of proteins, and this value was approximately 25-fold higher than that of the parent drug, metformin. Compound **2** was characterized by a moderate affinity (Table 1) towards OCT transporters; therefore, we presume that this compound might be transported with the aid of a transporter other than OCT, which is present mainly in MCF-7 but not in MDA-MB-231 cells, such as PMAT (Supplementary Figure S1). This statement could be confirmed by relatively low uptake of compound **2** in MDA-MB-231 cells, which demonstrated over three-fold lower PMAT expression. In turn, compound **3** was transported into MCF-7 and MDA-MB-231 cells at a comparable rate (0.84 ± 0.06 nmol/min/mg proteins and 0.42 ± 0.15 nmol/min/mg protein), and it was characterized by a low affinity towards OCTs. Thus, the compound possibly uses another transporter mechanism.

In the case of sulfonamides with bromide substituent in the aromatic ring, a similar “pattern” of uptake to chloride sulfonamides was reported for compounds **5** and **6**. On the other hand, compound **4** was transported into MDA-MB-231 cells approximately 130-fold more efficiently than in MCF-7. This phenomenon might be caused by a relatively high affinity towards OCTs in MDA-MB-231 cells (IC_50_ = 919.60 ± 13.0 μmol/L) and much higher OCT3 expression in these cells in comparison to MCF-7 [13]. However, it should be stated that the measured expressions were only at the RNA level. Thus, further proteomic studies are needed.

Derivatives with fluorine substituent in the aromatic ring were characterized by a greater uptake in MCF-7 cells than in MDA-MB-231 cells. For instance, the uptake rate of compound **7** was 1.592 ± 0.943 nmol/min/mg protein in MCF-7 cells, while in MDA-MB-231, it was 0.110 ± 0.01 nmol/min/mg protein. Compound **7** possesses a low affinity towards OCTs in both cell lines; therefore, we presume it might utilize another transporter mechanism, including PMAT. The most curious results were obtained for compound **9,** which was characterized by a quite high affinity towards OCTs (Table 1) in both cell lines. However, its uptake was moderate (0.357 ± 0.112 nmol/min/mg protein) in MCF-7 and very low in MDA-MB-231 cells (0.021 ± 0.002 nmol/min/mg proteins). We presume that this phenomenon might stem from the higher affinity for MATE transporters, which might work in combination with OCTs and mediate the elimination of this compound outside the cells, since they also serve as efflux transporters [18].

#### 2.3.2. Kinetic Analysis of Sulfonamide Uptake in MCF-7 and MDA-MB-231 Cells

The first stage of analysis consisted of determination of the relationship between the concentration of the test compound and its uptake in cells and the analysis of obtained Michaelis-Menten curves. The results of the kinetic parameters of the received curves are presented in Supplementary Table S1. In several cases, the Km and Vmax parameters could not be calculated, since the uptake was linear, and no transporter saturation was observed over the entire concentration range. The cases in which an analysis of the kinetic parameters was possible allow us to conclude that intracellular transport in the MCF-7 cell line was more effective than in MDA-MB-231, since the Vmax/Km ratios, corresponding to uptake efficacy, were higher in MCF-7 cells.

The in-depth analysis included the transformation of the obtained curves into Eadie-Hofstee plots, and then the subsequent calculation of Km and Vmax values. In addition, Vmax/Km ratios (mL/(min·mg)), which generally correspond to the intrinsic clearance and provide a link between transporter kinetics and in vivo pharmacokinetic variables, [19] were calculated. Thus, the Vmax/Km ratio corresponds to the efficacy of the transporters. A summary of the obtained results is presented in Table 2.

**Figure 3:**
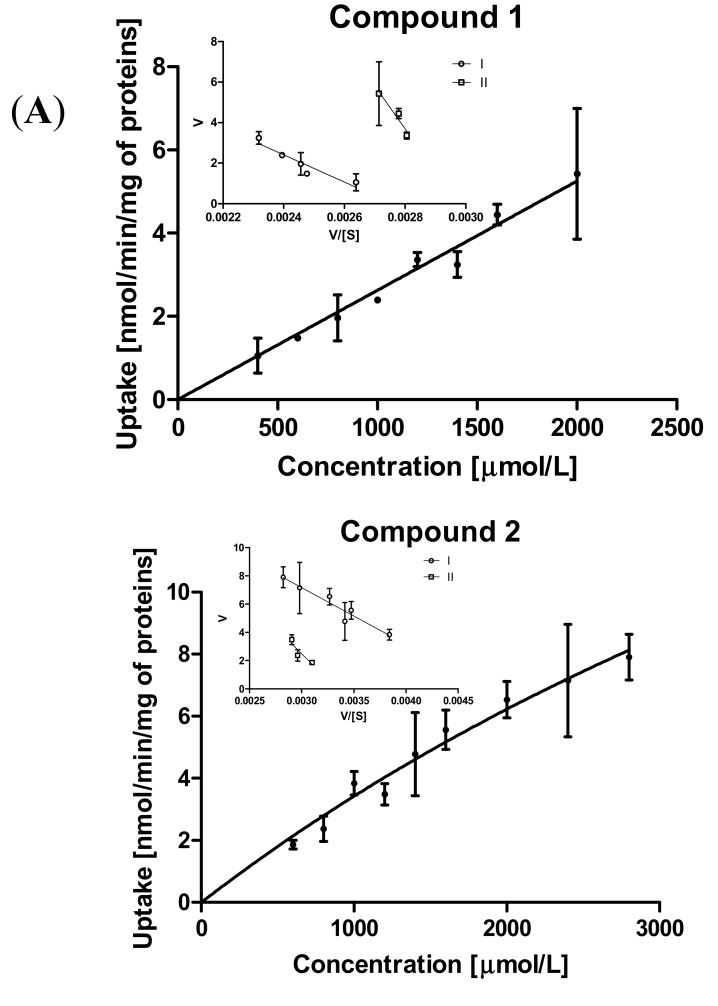

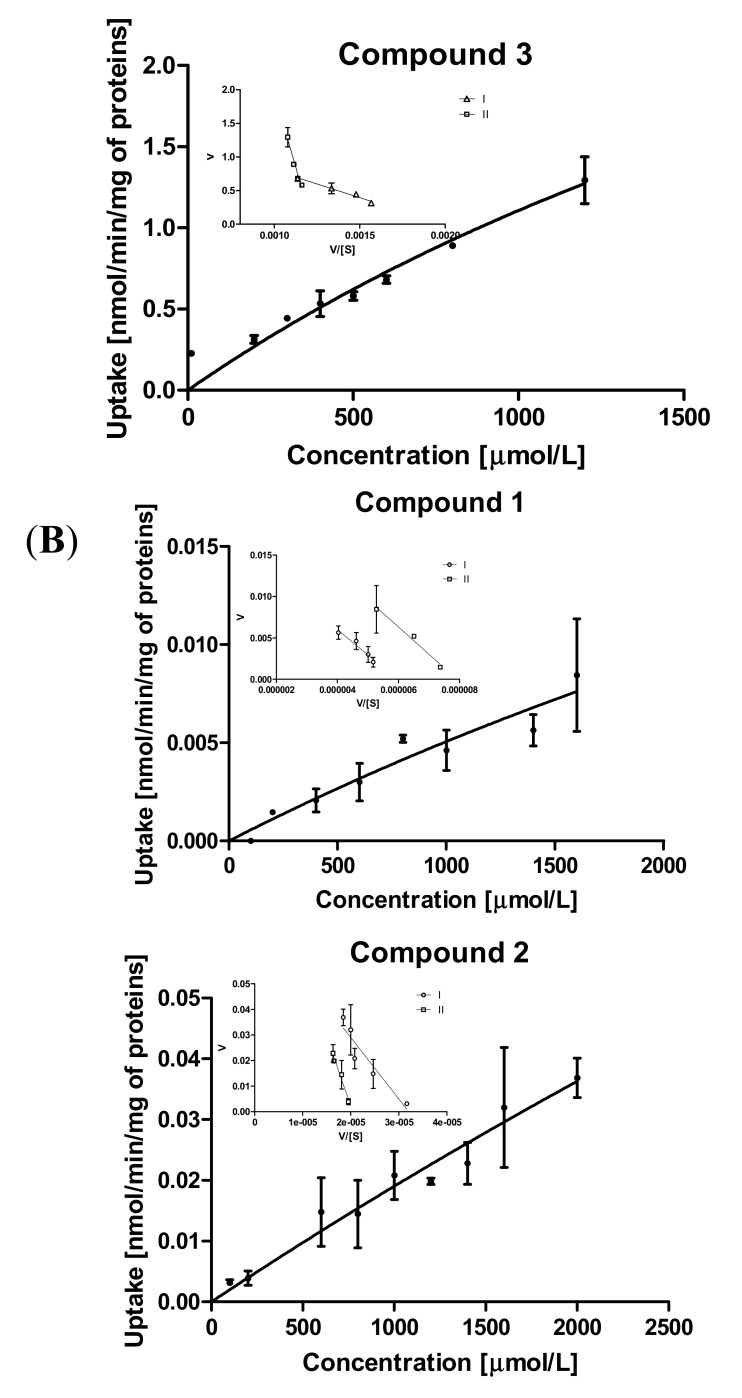

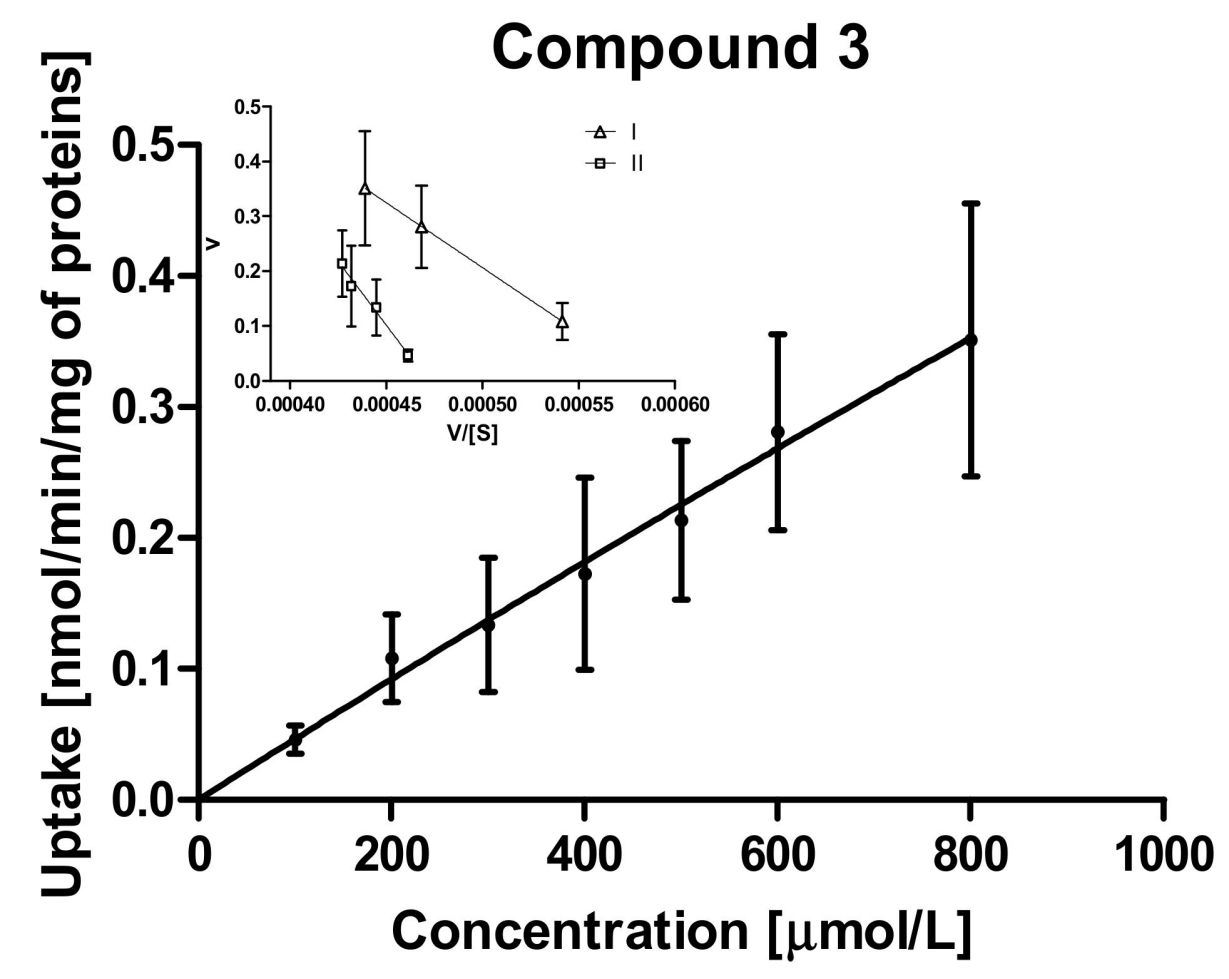
(**A**) The uptake of chloro-benzenesulfonamides (**1**–**3**) into MCF-7 cells at the concentrations of 10–3000 µmol/L and Eadie–Hofstee plots for organic cation transporters’ (OCTs’) mediated transport. (**B**) The uptake of chloro-benzenesulfonamides (**1**–**3**) into MDA-MB-231 cells at the concentrations of 10–2000 µmol/L and Eadie–Hofstee plots for OCTs’ mediated transport.

By taking into account the uptake curves of chloro-substituted sulfonamides in MCF-7 and MDA-MB-231 cells (Figure 3A,B), we can conclude that uptake of *ortho*- and *meta*-derivatives is much more efficient in MCF-7 cells. The Eadie-Hofstee analysis of metformin uptake showed that two transporters (marked as I and II in Figure 3A,B and Table 2) are engaged in the cellular uptake of each compound in both cell lines.

According to the Vmax/Km ratios of compounds **1**–**3** presented in Table 2, both transporters in MCF-7 cells appeared to be more efficient than in MDA-MB-231 cells. However, the most significant differences can be observed for compounds **1** and **2**. Both transporters in MCF-7 cells carrying compound **1** into the cells appear to be low-affinity high-capacity transporters, since the respective Km and Vmax values were relatively high (Table 2). In turn, both transporters in MDA-MB-231 cells carried derivative **1** into the cells at a low rate (low-capacity transporters), which was confirmed by an over 370-fold lower transport efficacy (lower Vmax/Km ratio). Compounds **1** and **2** were uptaken in both cell lines in a similar manner (Table 2 and Figure 3A,B). In contrast, one of the transporters in MCF-7 carrying compound **3** was a high-affinity medium-capacity transporter (Km = 812.7 μmol/L; Vmax = 1.61 nmol/min/mg proteins). The second transporter behaved more like a low-affinity high-capacity transporter. Curiously, compound **3** was uptaken into MDA-MB-231 cells also quite efficiently. However, Vmax/Km ratios were 2.4–3.2-fold lower than in MCF-7 cells. According to the Km and Vmax values, it appears that, in the MDA-MB-231 cell line, both transporters participate equally in the transport of compound **3**.

**Figure 4:**
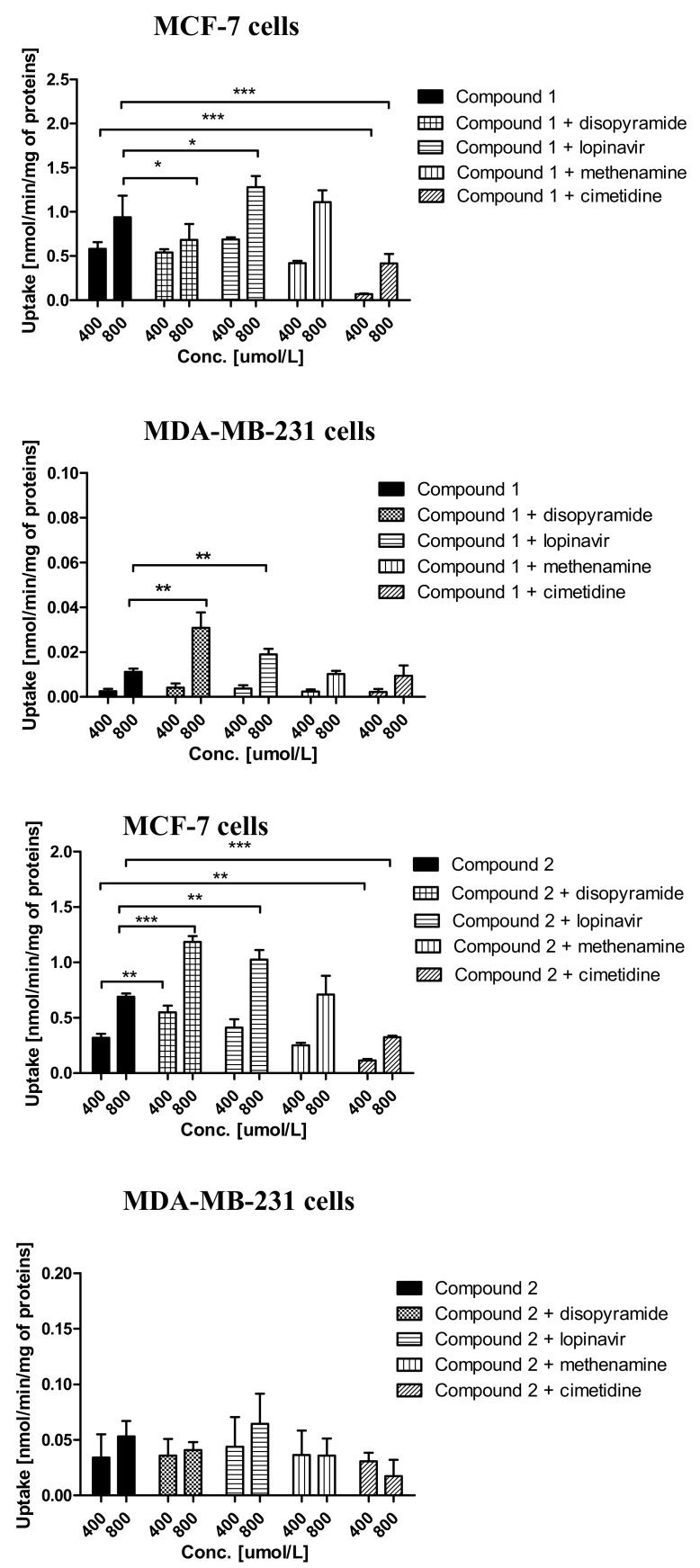

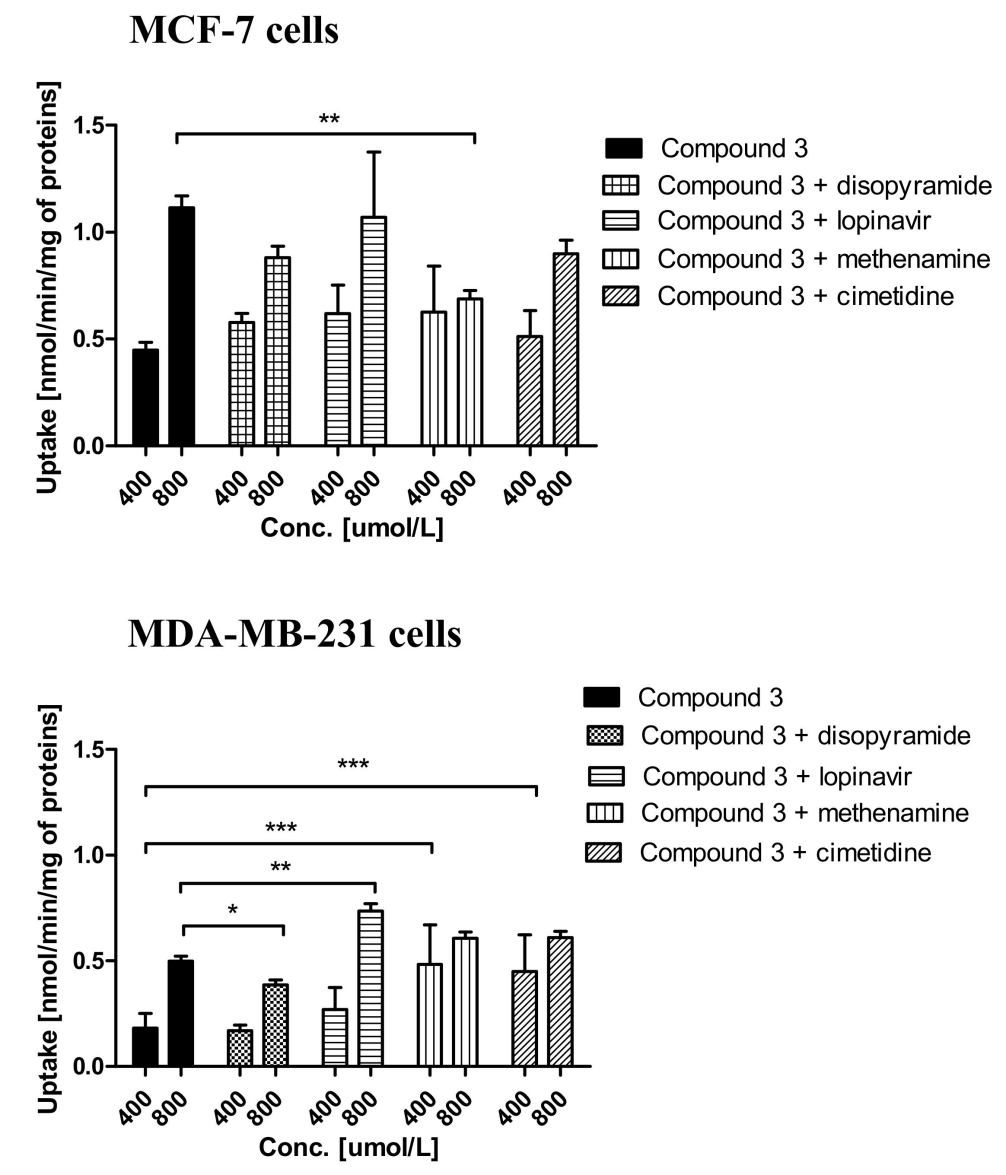
The uptake mechanism of compounds **1**–**3** (400 and 800 µmol/L) into MCF-7 cells and MDA-MB-231 cells. The uptake was determined in the presence of OCT and MATE inhibitors, disopyramide, lopinavir, methenamine or cimetidine (400 and 800 µmol/L) for 10 minutes at 37 °C. One-way ANOVA analysis was performed to compare the uptake of pure compounds (**1**–**3** at 400 and 800 µmol/L) with their uptake in the presence of transporter inhibitors. The significant differences between the uptake of pure compounds **1**–**3** (black bars) and their respective mixtures with inhibitors (disopyramide, lopinavir, methenamine or cimetidine) are marked with black lines and are denoted with asterisk. * *p* < 0.05, ** *p* < 0.01 and *** *p* < 0.001. In MCF-7 cells, the uptake of compound **1** at 400 µmol/L was significantly decreased in the presence of cimetidine, while at 800 µmol/L, the significant differences were reported in the presence of disopyramide, lopinavir and cimetidine. In MDA-MB-231 cells, the uptake of compound **1** at 800 µmol/L was significantly increased in the presence of disopyramide and lopinavir. The significant differences in the uptake of compound **2** were reported in the presence of disopyramide, lopinavir and cimetidine in MCF-7 cells, while in MDA-MB-231 cells, no significant changes in the uptake of **2** in the presence of all inhibitors were reported. In MCF-7 cells, the uptake of compound **3** at 800 µmol/L was significantly decreased in the presence of methenamine in comparison to compound **3** alone. In MDA-MB-231 cells, the uptake of compound **3** was significantly different in the presence of all inhibitors, depending on the compound concentration.

To elucidate which transporters are engaged in the cellular uptake of sulfonamides, the experiments with OCTs and MATE inhibitors were performed. The effects of a few transporters’ inhibitors on the uptake of chloro-benzene-sulfonamides are presented in Figure 4. The uptake of compound **1** in MCF-7 cells was significantly decreased only in the presence of methenamine and cimetidine, which suggests that MATE1 and OCT3 transporters, respectively, might participate in the cellular uptake of **1**. Lower uptake, however, not statistically significant, was also reported in the presence of disopyramide (Figure 4), an OCT1 inhibitor.

Co-treatment of MCF-7 cells with compound **1** and lopinavir showed a significantly higher uptake of **1**, which indicates that either another low-affinity high-capacity transporter is used when PMAT is inhibited or PMAT is transporting compound **1** out of the cell as an efflux transporter. In the case of MDA-MB-231 cells, a significantly higher uptake of compound **1** in the presence of both disopyramide and lopinavir was observed (Figure 4), implying that compound **1** can utilize some other higher capacity transport mechanism when OCT1 is inhibited and that it also has affinity for PMAT. No statistically significant changes in the uptake of **1** in the presence of both methenamine and cimetidine was reported in MDA-MB-231 cells, which suggests that OCT3 and MATE1 are not responsible for a weak uptake of compound **1** in MDA-MB-231 cells. The uptake of compound **2** exhibiting moderate affinity towards OCTs in MCF-7 cells (Table 1) was profoundly elevated in the presence of both disopyramide and lopinavir, similar to compound **1** in MDA-MB-231 cells, and inhibited by cimetidine (Figure 4). In MDA-MB-231 cells, no significant differences were observed in the uptake of **2** in the presence of all inhibitors (disopyramide, lopinavir, methenamine and cimetidine). However, a slightly decreased uptake of **2** was found in the presence of disopyramide and cimetidine, which suggests that OCTs may be the low-affinity low-capacity carriers responsible for the intracellular transport of this compound in MDA-MB-231 cells. The uptake of compound **3** did not change in the presence of disopyramide, lopinavir and cimetidine in MCF-7 cells. A significant decrease in the uptake of compound **3** was reported in the presence of methenamine, which suggests that MATE1 transporters participate in its uptake in MCF-7 cells. In MDA-MB-231 cells, we reported a significantly lower uptake of compound **3** at 800 µmol/L in the presence of disopyramide, so we can deduce that OCTs, presumably OCT1, participate in the transport of this compound. Additionally, increased uptake of **3** was reported in the presence of lopinavir and methenamine, which implies a potential interaction with PMAT and MATE. The summary of possible interactions of studied compounds with transporters is presented in Supplementary Table S2.

A completely different cell uptake model was shown for compound **4**, which was uptaken more efficiently in MDA-MB-231 cells than in MCF-7. This fact is manifested with higher respective Vmax values and Vmax/Km ratios than most of the other compounds (Figure 2 and Table 2). In MDA-MB-231 cells, both transporters appear to be equally efficient in the transport of compound **4** and could be regarded as low-affinity high-capacity transporters. Another analyzed derivative, **5**, was characterized by moderate uptake. Yet, it was still better uptaken in MCF-7 cells than metformin. The uptake of compound **6** with a *p*-bromo substituent was good and reached values of 1.11 ± 0.16 and 0.75 ± 0.29 nmol/min/mg of proteins in MCF-7 and MDA-MB-231 cells, respectively. Based on an uptake comparison of both these derivatives in MCF-7 and MDA-MB-231 cells, we can conclude that the uptake of both derivatives is better in MCF-7 cells (Figure 2). However, the differences are more pronounced in compound **5**. The Eadie-Hofstee analysis of *m*-, and *p*-bromo derivatives’ (compounds **5** and **6**) uptakes showed two transporters engaged in the cellular uptake of compounds in both cell lines (Table 2). In the case of compound **5**, both transporters appeared to be more efficient in MCF-7 cells than in MDA-MB-231 cells (higher Vmax/Km ratios). However, this derivative seems to possess a higher affinity towards the transporters in MDA-MB-231 cells, as the respective Km values were lower (Table 2). Both transporters in MDA-MB-231 cells behaved more like higher-affinity lower-capacity for **5**, while in MCF-7, they had a lower affinity and possessed greater capacity. The Eadie-Hofstee analysis showed a strong affinity of compound **6** towards one transporter in the MCF-7 cells with Km = 307.6 μmol/L. The other transporter behaved more like a low-affinity high-capacity carrier (Table 2). In contrast, compound **6** exhibited comparable affinities towards both transporters in the MDA-MB-231 cells.

The uptake of compounds **4**–**6** was also studied in the presence of OCT inhibitors (Supplementary Figure S2. In MCF-7 cells, the inhibitors did not change the weak uptake of compound **4**, except for lopinavir and some extent with disopyramide, which, similarly to the aforementioned chloro derivatives, increased the uptake of **4**. In MDA-MB-231 cells, the relatively high uptake of **4** was significantly decreased in the presence of disopyramide and cimetidine but increased in the presence of lopinavir, which suggests that OCT 1 and 3 participate in its uptake, and it can interact with PMAT. Compounds **5** and **6** were transported efficiently in MCF-7 cells (Figure 2), and their uptake was significantly reduced when cotreated with disopyramide. The uptake of compound **5** also increased in the presence of lopinavir and methenamine (Supplementary Figure S2), which indicates the interactions of compound **5** with PMAT and MATE1. In addition to OCT1, compound **6** utilizes also PMAT and MATE, since the uptake of compound **6** was significantly decreased in the presence of lopinavir and methenamine (Supplementary Figure S2). The effective uptake of compound **6** in MDA-MB-231 cells was significantly decreased in the presence of disopyramide, methenamine and cimetidine (Supplementary Figure S2), suggesting the participation of OCT1 and 3 and MATE1.

Based on uptake comparisons of fluorinated sulfonamides (compounds **7**–**9**) in MCF-7 and MDA-MB-231 cells, we can conclude that the uptake was much better in MCF-7 (Figure 2). Eadie-Hofstee analysis of *o*-fluoro-benzenesulfonamide (compound **7**) showed 2 transporters engaged in cellular uptake of the derivative in both cell lines. Both transporters in MCF-7 cells appeared to be 10-fold more efficient than in MDA-MB-231 cells and can be regarded as moderate-affinity high-capacity transporters (Table 2). In MDA-MB-231 cells, derivative **7** seemed to have a moderate affinity for both transporters; however, their capacity was low. Therefore, a moderately low uptake comparable with parent drug metformin in these cells was observed.

Based on the obtained results (Table 1 and Table 2), it can be concluded that *m*- and *o*-fluoro benzenesulfonamides (**8** and **9**) are not good substrates for any of the recognized transporters in either cell line. In MCF-7 cells, compound **8** presented two-phase uptakes (Supplementary Figure S3. Up to the concentration 1200 μmol/L, the uptake was moderate and proceeded linearly, while at higher concentrations (above 1600 μmol/L), the uptake was extremely high, reaching 8–10 nmol/min/mg of protein. This behavior was reflected in the results of the Eadie-Hofstee analysis, which revealed that two transporters are engaged in the uptake of compound **8** in MCF-7 cells. One transporter responsible for the uptake of compound **8** at lower concentrations was acting as a moderate-affinity moderate-capacity transporter (Table 2 and Supplementary Figure S3). At higher concentrations exceeding 1600 μmol/L, compound **8** favored the other transport mechanism. However, we presume that the obtained results might be associated with a strong, direct effect of this compound on the integrity of the cellular membrane. In contrast, the uptake of compound **8** in MDA-MB-231 cells was much lower (Figure 2), which might be explained by the approximately 22-fold lower transport efficiency (Vmax/Km) in MDA-MB-231. Compound **9** presented a similar uptake profile in MDA-MB-231 cells, with two low-affinity low-capacity transporters carrying it into the cells. In the case of MCF-7 cells, compound **9** presented a biphasic (sinusoid) uptake and appears to use its own mechanism, which might stem from its interaction with the cell membrane.

The uptake of compounds **7**–**9** in the presence of transport inhibitors is presented in Supplementary Figure S4. Uptake in MCF-7 cells of these fluoro derivatives was not changed in the presence of disopyramide; however, in the case of compound **7** a decrease in uptake in the presence of cimetidine was observed. Therefore, it can be stated that OCT3 participates in compound **7** uptake in MCF-7 cells. Compound **7** may also have an affinity for PMAT, because an increase in its uptake was reported in the presence of lopinavir. Unfortunately, due to the high variation, the changes were not statistically significant. In turn, the uptake of **7**–**9** in MDA-MB-231 was increased in the presence of both disopyramide and cimetidine (Figure S4). The increased uptake of **7**–**9** might be explained by the fact that OCT inhibitors hindered the studied compounds to bind to OCTs and forced them to use other high-capacity transporters.

In conclusion, this study is the first one to demonstrate how the chemical transformation of the biguanide backbone into sulfonamides differing with a halogen substituent in the aromatic ring affects the intracellular transport in two commonly studied unmodified human breast carcinoma cell lines, MCF-7 and MDA-MB-231. The cell lines were chosen on the basis of a comprehensive study of Cai et al. [14], who characterized the expression of OCTs, PMAT and MATE transporters in several commonly used human breast cancer cell lines. MCF-7 cells were found to express mainly PMAT and MATE transporters; however, OCT1 was also present. In the case of MDA-MB-231 cells, OCT3 and MATE were found. The characterization of transporters’ expressions in various cell lines allowed the authors to conclude that there is a relationship between the presence of cation-selective transporters, the effectiveness of metformin uptake and its antiproliferative activity [14]. In addition, the authors assessed the expression of OCT, PMAT and MATE transporters in human breast tissues. Importantly, OCT3 and PMAT were the predominant transporter genes expressed in normal and breast tumor tissue, while the OCT1 and MATE1 genes showed lower expression rates. The researchers compared also metformin uptake and antiproliferative activity between a cation-selective transporter-deficient human breast cancer cell line, BT-20, and a BT-20 cell line that was engineered to overexpress OCT3, a representative of cation-selective transporters and a predominant transporter in human breast tumors. The scientists found that metformin uptake was minimal in BT-20 cells but increased by more than 13-fold in OCT3-BT20 cells [14]. Additionally, our previous paper [13] revealed that metformin was uptaken more efficiently in MDA-MB-231 cells than MCF-7 cells, and it was correlated with a greater expression of OCT3 in MDA-MB-231 cells. Based on these results, we decided to conduct the studies using two commonly used breast cancer cell lines differing in the expression of cation-selective transporters. 

A comparison of the uptake profile of all halogenated derivatives allows us to conclude that most of the compounds express a higher affinity for OCTs and are more efficiently uptaken in MCF-7 cells than MDA-MB-231 cells. Compound **4** with a *o*-bromo substituent which showed greater uptake in MDA-MB-231 cells than MCF-7 and, thus, is the most prominent exception in this respect. Decreased uptake of **4** in the presence of OCT inhibitors, disopyramide and cimetidine suggests participation of these transporters in its uptake in MDA-MB-231 cells. By comparing the groups of compounds concerning the halogen substituent, we can conclude that the presence of a chlorine atom in the aromatic ring contributes to the largest uptake in MCF-7 cells. Substantially lower uptakes of **1**–**3** in MDA-MB-231 cells result from a lower affinity towards their transporters and lower capacity of the transporters, expressed as the decreased Vmax/Km ratio (Table 2). Among compounds containing chlorine and bromine substituents, we can observe that these substituents in the *para* position in the aromatic ring (compounds **3** and **6**) contribute to a relatively good uptake in both cell lines. Furthermore, the “addition” of a fluorine substituent in the aromatic ring in sulfonamide derivatives contributed to a substantial decrease in the affinity for OCTs, especially in the *o*- and *m*- positions and concomitant reduction in cellular uptake mainly in MDA-MB-231 cells. Based on the uptake studies in the presence of inhibitors, we presume that compounds with *o*- and *m*-chloro- and bromo-substituents, as well as the *o-*fluoro substituent, can use not only OCT transporters but also PMAT and/or MATE in MCF-7 cells, and therefore, they are more efficiently uptaken. On the other hand, in MDA-MB-231 cells, fluorine compounds were uptaken mainly with the aid of OCT1 and OCT3 transporters.

Another important issue when discussing obtained results and differences in the cellular uptake of tested sulfonamides in MCF-7 and MDA-MB-231 cells is the estrogen receptor status (ER). MCF-7 cells are luminal breast cancer that expresses estrogen receptors (ERs), while MDA-MB-231 cells are triple-negative basal-like breast cancer cells. Based on this different cell origin and ER expression, we cannot exclude the possible role of ER in the uptake of studied compounds. Therefore, the possible role of ER OCT1-3, PAMT and MATE expression and function should be examined attentively in the future, since there is no scientific data regarding the role of ER receptors in the cellular uptake of metformin. The most comprehensive studies have been published by Cai et al. [14] who have characterized the expression of OCT, PMAT and MATE transporters in several commonly studied human breast cancer cell lines, including MCF-7 and MDA-MB-231 cells, and correlated it with antiproliferative properties of metformin. The currently ongoing studies have focused mainly on the role of ER in multidrug resistance mainly through efflux transporters, including P-glycoprotein, multidrug resistance-associated protein (MRP) or breast cancer resistance protein (BCRP), which is a major obstacle in the effective therapy of breast cancer [20]. For instance, Chang et al. [21] found that estrogen induces the expression of multidrug transporter gene ABCG2 (BCRP) through ERα, making MCF-7 more tolerant to mitoxantrone. Previously, it was also found that estrogen stimulates SLC22A5 (organic cation transporter, OCTN2) expression strongly in an ER-dependent manner and that SLC22A5 expression is associated with the ER status in breast cancer cell lines and breast tissue [22]. These results are important, since SLC22A5 is required for carnitine intake, lipid metabolism and cell proliferation. However, according to these papers, ER is only a gene regulator of BCRP and OCTN2, which can increase the expression and, subsequently, function of the transporter. It is important to keep in mind that transporter regulation consists also of other steps, including post-translational modification and transport and anchoring the protein to the plasma membrane, and therefore, transporters’ expression modulation, meaning upregulation or downregulation, is a multistep process consisting of several different factors that all need to be studied very carefully [23].

Bearing in mind conclusions made by Cai et al. [14] regarding the greater anticancer effects of metformin correlating with its higher uptake, we presume that an increased uptake of the examined sulfonamide derivatives of metformin may translate into a greater potency of the biological activity, including the antiproliferative effect.

### 2.4. Cell Viability Assay

Taking into account the above-mentioned findings, we decided to check if the chemical modification of the metformin structure and subsequent higher cellular uptake can affect the biological response of the cells. Cell viability was the basic parameter that we determined. Effects of halogenated sulfonamide derivatives of metformin on the viability of MCF-7 and MDA-MB-231 cells were analyzed using the WST-1 assay, which proves mitochondrial function. 

In the case of MCF-7 cells, for all studied compounds, a concentration-response analysis to determine the concentrations inducing a 50% decreased cell viability (IC_50_) was performed (Table 3). 

The cells were stimulated with sulfonamides **1**–**9** at concentrations of the same range as in uptake studies (10 µmol/L to 3000 µmol/L). Compound **1** with an *o*-chloro substituent in the aromatic ring was found to be the most cytotoxic towards MCF-7 cells. Its IC_50_ value was 12.6 ± 1.2 µmol/L. Additionally, *o*-fluoro derivative (**7**) presented high cytotoxic properties in MCF-7 cells. It might be expected that relatively low IC_50_ values and profound cytotoxic properties of both these compounds result from their high uptake in MCF-7, which was approximately 14-fold higher than that of metformin in MCF-7 cells. In turn, the uptake of compounds **1** and **7** in MDA-MB-231 cells was much lower and did not exceed 0.1 nmol/min/mg of protein, and as a consequence, the properties towards the inhibition of cell growth were significantly weaker. For instance, compound **1** at 3000 µmol/L contributed to 69.25 ± 12.11% viability of MDA-MB-231. For compound **7,** the IC_50_ value of MDA-MB-231 growth inhibition was 2594 ± 150 µmol/L. A similar tendency towards a more effective growth inhibition of MCF-7 cells in comparison to MDA-MB-231 was observed for most of the other tested compounds. However, the opposite trend was observed in the case of compound **4** with the *o*-bromo substituent. This compound was weakly uptaken in MCF-7 cells, but its IC_50_ value of MCF-7 viability is 429.7 ± 119.0 µmol/L. Adversely, the uptake in MDA-MB-231 cells was 1.3 ± 0.21 nmol/min/mg protein, and compound **4** at 3000 µmol/L inhibited cell viability approximately up to 56.4%. This phenomenon might stem from the fact that compound **4** is not so potent, and it was not transported as efficiently into the cells as the others.

Compound **3** was characterized by a quite high cellular uptake in both cell lines, which was reflected by moderate cytotoxic properties (IC_50_ values 1430 ± 135.0 and 1042 ± 134.0 µmol/L for MCF-7 and MDA-MB-231 cells, respectively). Furthermore, compound **6** was uptaken in MCF-7 and MDA-MB-231 cells at a comparable rate. However, they showed a different potential towards cell growth inhibition, since, in MCF-7 cells, compound **6** decreased viability of 50% of the cells at 1142 ± 139.0 µmol/L, while, in MDA-MB-231, the IC_50_ value was 2106 ± 137.0 µmol/L.

**Figure 5:**
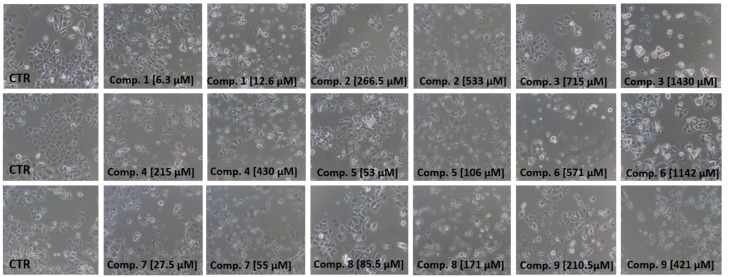
Dose-dependent effects of sulfonamide derivatives of metformin (compounds **1**–**9**) at concentrations corresponding to their ½ × IC_50_ and IC_50_ (µmol/L) values on MCF-7 cells’ viability and morphology after 24 h of incubation. Representative cell images are shown (100-fold magnification); CTR—control.

**Figure 6:**
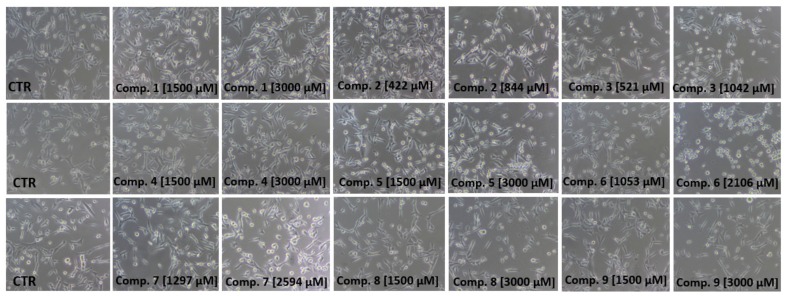
Dose-dependent effects of sulfonamide derivatives of metformin (compounds **1**–**9**) on MDA-MB-231 cells’ viability and morphology after 24 h of incubation. Compounds **2**, **3**, **6** and **7** were used at concentrations corresponding to their ½ × IC_50_ and IC_50_ (µmol/L) values. Compounds **1**, **4**, **5**, **8** and **9** decreased the viability of MDA-MB-231 cells to approximately 60%. Their effects on MDA-MB-231 cells’ morphology were evaluated using the concentrations of 1500 and 3000 µmol/L. Representative cell images are shown (100-fold magnification); CTR—control.

The effects of examined sulfonamides **1**–**9** on MCF-7 and MDA-MB-231 cells were also evaluated using light and phase-contrast microscopy studies. As presented in Figure 5 and Figure 6, sulfonamides exerted a diversified influence on the morphology of both cell lines. Images in Figure 5 show that sulfonamides at concentrations corresponding to their ½ × IC_50_ and IC_50_ values contributed to a decrease in the number of viable cells, their density and alteration in MCF-7 cells’ morphology. In contrast, the studied compounds at higher concentrations—that is, up to 3000 µmol/L—did not contribute to such extensive changes in the viability and density of MDA-MB-231 cells (Figure 6), particularly in the case of compounds **1**, **4**, **5**, **8** and **9**. For instance, MDA-MB-231 cells treated with compound **1** at concentrations of 1500 µmol/L and 3000 µmol/L did not exhibit any profound morphological changes. However, the greater number of rounded cells, membrane disruption and inhibition of growth were reported. Simultaneously, in the case of MCF-7 cells, after 24 h of cell co-treatment with **1** at a much lower concentration (12.6 µmol/L), severe compound-mediated changes, manifested by membrane disruption, cell shrinkage, rounding and inhibition of growth, were observed (Figure 5). In summary, the microscopy studies confirm results obtained in the viability assay, since most of the analyzed compounds exhibited more profound growth inhibitory properties in MCF-7 cells than in MDA-MB-231 cells.

Aforementioned results indicate that chemical modification of the metformin backbone into halogenated sulfonamides leads to obtaining compounds demonstrating much higher cytotoxic properties than those of the parent drug, metformin, which, as it was previously found, did not significantly affect MCF-7 and MDA-MB-231 growth up to 3000 µmol/L [13]. These promising results might stem from a greater affinity towards OCTs and higher cellular uptake, especially in MCF-7 cells. This statement seems to be supported by Cai et al. [14], who correlated transporter expression profiles with the cellular uptake and antiproliferative activity of metformin. The authors found that metformin uptake in OCT3-BT20 cells was over 13-fold higher compared to uptake in BT-20 cells. Analogous conclusions were presented by Checkley et al. [17], who found that intratumoral metformin accumulation highly correlates with OCT2 expression. Based on the literature review [14] and our earlier experience [13], we presume that metformin uptake and its antiproliferative effects in breast cancer cell lines is transporter-dependent.

Bearing in mind the results of the current study, we can conclude that chemical modification of the biguanide scaffold into sulfonamides with the halogen-substituted aromatic ring results in greater uptake and subsequent antiproliferative activity, particularly in MCF-7 cells. We are aware that a particular compound that exerts cytotoxic properties towards cancer cell lines may also be toxic towards primary cells. Therefore, in our previous paper, we published the results of viability studies using human endothelial cells (human umbilical vein endothelial cells, HUVECs) and aortal smooth muscle cells (AoSMC). They showed that most halogenated compounds weakly inhibit the growth of primary cells, since at 3000 µmol/L, the compounds decreased the viability of HUVECs and AoSMCs to 65–80%. Within the concentration range 10–3000 µmol/L, the IC_50_ values were calculated only for compound **3.** They appeared to be 1770 ± 120 µmol/L, and 1890 ± 110 µmol/L in HUVECs and AoSMCs, respectively [24]. Collectively, the chemical transformation of metformin into halogenated benzenesulfonamides might be regarded as a novel solution for the improvement of metformin cytotoxicity.

### 2.5. Apoptosis Assay

Taking into consideration the differential affinity for OCTs’ uptake in MCF-7 and MDA-MB-231 cells and subsequent inhibition of cell growth, we designated three compounds for further studies. Since the inhibition of cell proliferation may lead to the initiation of apoptosis, we decided to stain the cells with annexin V and propidine iodide and determine whether sulfonamides with halogen atoms in *ortho* position in the aromatic ring (compounds **1**, **4** and **7**) affect breast cancer cells’ growth by an induction of apoptosis.

Apoptosis is a fundamental biological process for the removal of unwanted cells, which is characterized by various biochemical changes, including the translocation of membrane phosphatidylserine (PS) from the inner side of the plasma membrane to the surface. Staining the cells with fluorochrome-labeled annexin V, a phospholipid-binding protein, enables to detect exposed PS using flow cytometry. Since propidine iodide is not permeant to live cells, it is commonly used to detect dead or late-apoptotic cells.

Results of the apoptosis assay showed that co-treatment of MCF-7 cells with compounds **1**, **4** and **7** contributed to a significant decrease in the number of single cells collected for analysis in gate B (Table 4).

**Figure 7:**
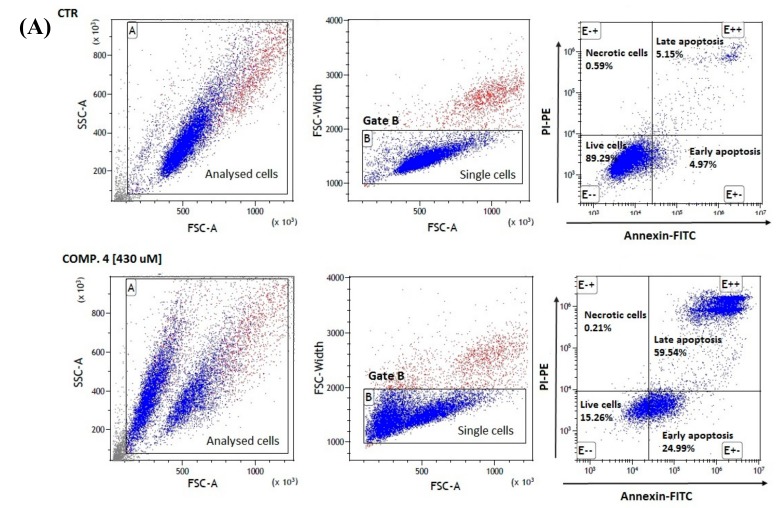

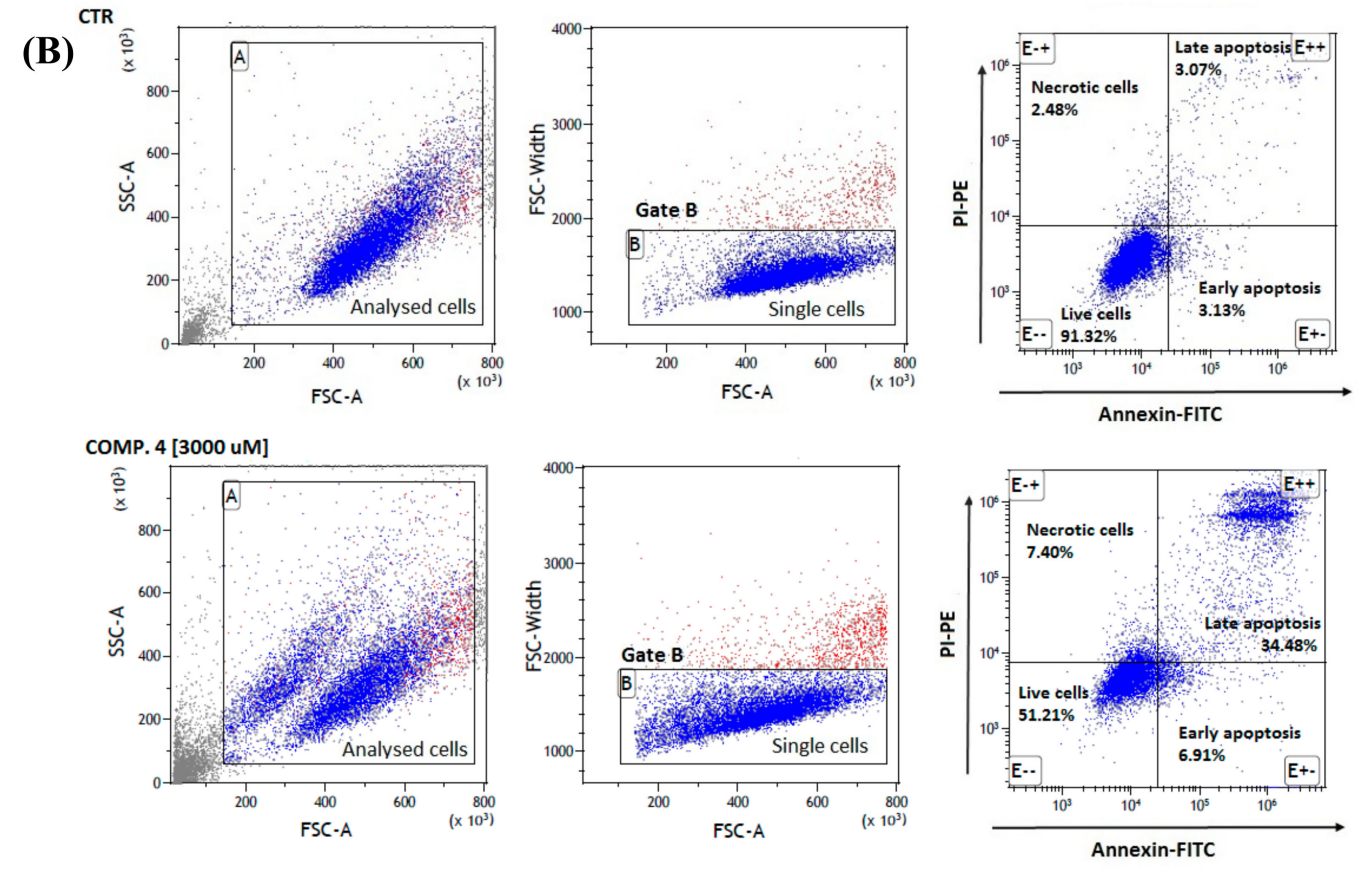
The effects of compound **4** on MCF-7 (**A**) and MDA-MB-231 (**B**) cells’ viability and apoptosis. The cytograms in the upper line are representatives for control cells; the cytograms in the lower line are representatives for compound **4**. Cytograms on the left side—forward and side scatter plot of MCF-7 cells. The cells were marked with gate A (the events not enclosed with the gate are smaller and might constitute some parts of broken cells (cell debris)). Cytograms in the centre—a forward scatter width (FSC-W) vs. forward scatter area (FSC-A) of the cells gathered in gate A. Based on FSC-A parameters, single cells were divided (in the lower parts of the cytogram) and marked with gate B. The single cells in gate B were analyzed for staining with annexin V and propidine iodide. Cytograms on the right side—annexin V (x-axis) vs. propidium iodide (y-axis) plots from the gated cells (B) show the populations corresponding to living cells (annexin V (−) and PI (−)) (E−−)—lower-left square, early-apoptotic cells (annexin V (+) and PI (−)) (E+−)—lower-right square, late-apoptotic cells (annexin V (+) and PI (+)) (E++)—upper-right square and necrotic cells (annexin V (−) and PI (+)) (E−+)—upper-left square.

The analysis of cells stained with annexin V and propidine iodide showed a significant decrease in the percentage of living cells (AV−PI−). For instance, compound **4** at 215 µmol/L corresponding to its ½ × IC_50_ value reduced the number of viable cells up to 34.17 ± 3.88% in comparison to control samples (88.59 ± 0.96%); *p* < 0.001. At the same time, compound **4** induced an increase in the percentage of apoptotic cells as compared to the control cells. It should be noted that compound **4** at 215 µmol/L contributed to the induction of 30.69 ± 3.01% of early-apoptotic cells (AV+PI−) and 34.69 ± 1.51% of late-apoptotic cells, while the amount of 430 µmol/L contributed to a higher percentage of late-apoptotic (AV+PI+) cells (60.16 ± 2.21%). The representative cytograms showing the effects of compound **4** on apoptosis induction are presented in Figure 7. Stimulation of MCF-7 cells with selected sulfonamides does not increase the percentage of necrotic cells (AV−PI+); moreover, a statistically significant decrease in this parameter has been observed (Table 4).

In contrast, stimulation of the cells with derivatives **1**, **4** and **7** at the concentration of 1500 or 1300 μmol/L did not affect MDA-MB-231 cells’ population in gate B. Higher concentrations of these compounds contributed to a slight, yet significant, decrease in the percentage of cells collected in gate B. A quantitative analysis of MDA-MB-231 cells after 24h exposure to 1500 μmol/L of compound **1** contributed to a significant decrease in the number of viable cells (81.55 ± 1.65% vs. 90.68% ± 1.37% for control). A further increase in a compound **1** dose (3000 μmol/L) more profoundly decreased the number of viable cells (53.83% ± 3.87%). All tested compounds were found to induce similar changes in the percentage of early- and late-apoptotic cells (Table 4). In contrast to MCF-7 cells, the results showed a 2-3-fold increase in the percentage of necrotic cells in response to the compound treatments as compared to the control cells.

Collectively, the results of the apoptosis assay confirm that compounds **1**, **4** and **7** at a definitely lower concentration induce early and late apoptosis in MCF-7 cells. These results confirm that the effective uptake of sulfonamide derivatives of metformin in MCF-7 cells correlates positively with their biological effects, including antiproliferative properties and the ability to apoptosis induction. In summary, the *ortho*-halogens can be put in order **1** > **7** > **4**, which is in line with uptake studies.

### 2.6. Cell Cycle Arrest

To further elucidate the mechanisms responsible for the antiproliferative effects of sulfonamide derivatives of metformin, we investigated their properties towards cell cycle arrest. The experiments were performed using PI-stained cells, and the separation of cells in the G0/1, S and G2/M phases was analyzed by flow cytometry. The analysis of the cell cycle progression is presented in Table 5. 

**Figure 8:**
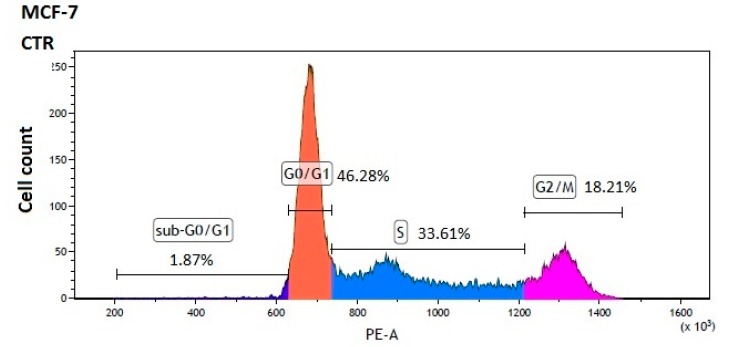

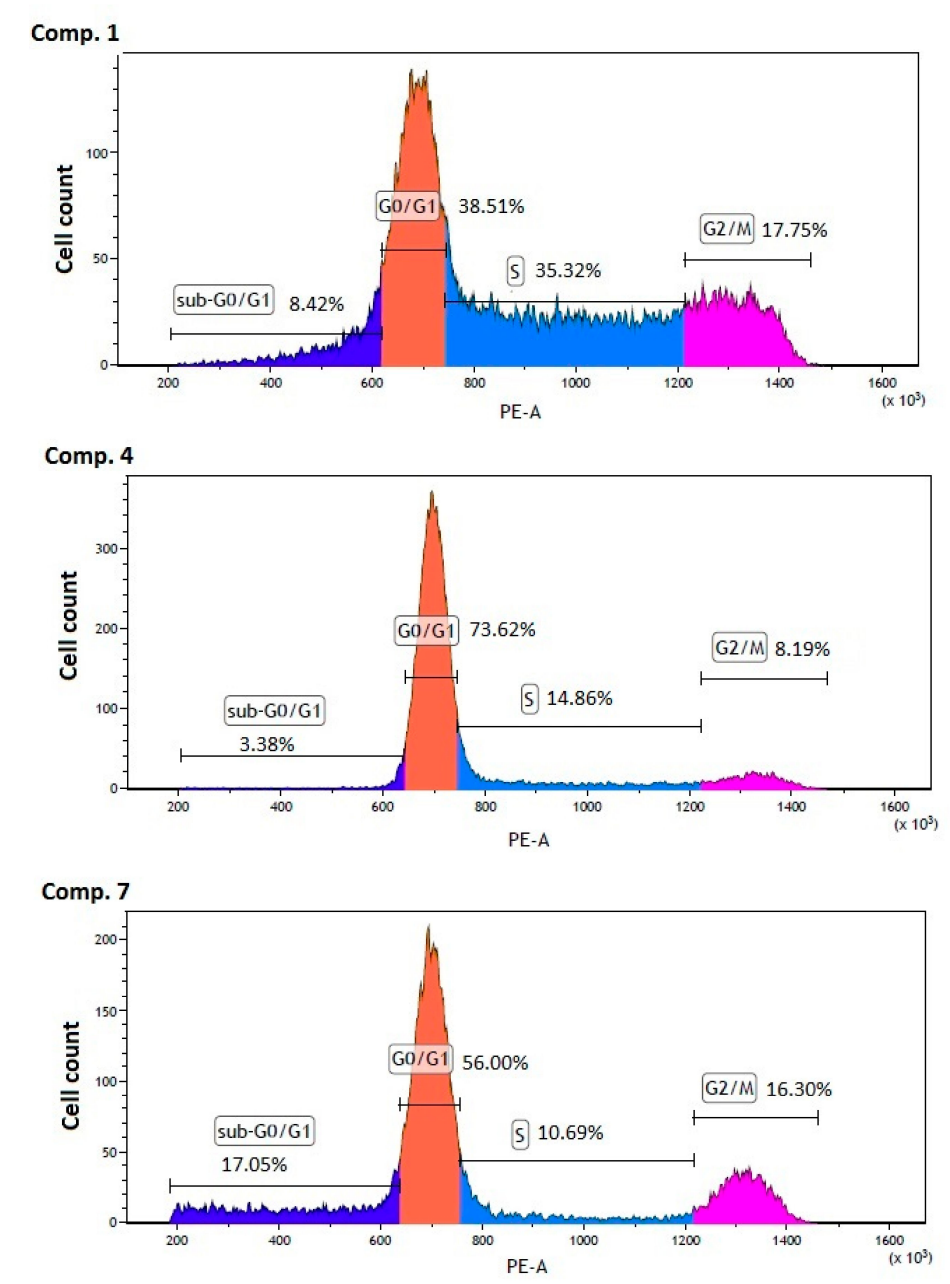

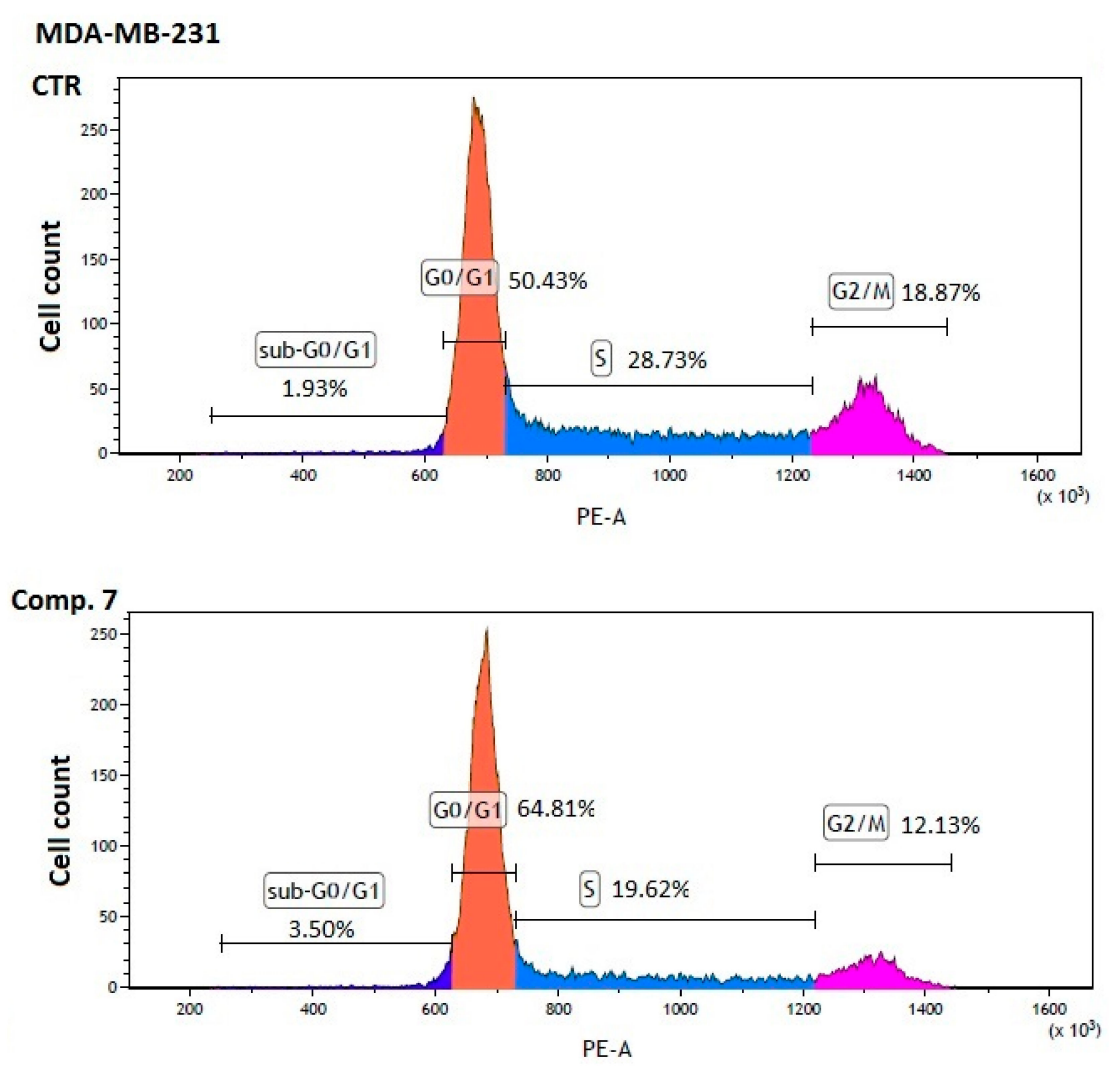
Effects of selected sulfonamides on MCF-7 and MDA-MB-231 cell cycle progression. MCF-7 cells were treated with compounds **1**, **4** and **7** at concentrations corresponding to their IC_50_ values for 24h. MDA-MB-231 were treated with compound **7** at a concentration corresponding to the IC_50_ value. DNA content was determined using a flow cytometry analysis of PI-stained cells. Representative cytograms of the cell cycle distribution at G0/1, S and G2/M phases are shown.

MCF-7 cells were treated with compounds **1**, **4** and **7** at a concentration equal to their IC_50_ values. Treatments of MCF-7 cells with compound **1** were associated with a significant decrease in the percentage of cells arrested at the G0/G1 phase (38.76 ± 0.80% vs. 45.56% ± 0.68% for control). However, the changes in S and G2/M populations, although statistically significant, might not be biologically effective. In the case of compound **4**, we found a significantly increased percentage of cells arrested at the G0/G1 phase with a concomitant decrease in the S and G2/M phases (Table 5). Compound **7** induced a similar response in MCF-7 cells; however, it also increased the percentage of cells in the sub-G0/G1 phase. Representative profiles of the cell cycle distribution of MCF-7 cells treated with compounds **1**, **4** and **7** are depicted in Figure 8.

MDA-MB-231 cells were treated with compound **7** at its IC_50_ value (2600 µmol/L), and the analysis of the cell cycle distribution showed a significant increase in the percentage of cells at sub-G0/G1 and G0/G1 phases and a reduced number of cell numbers in S and G2/M fractions (Table 5 and Figure 8).

To summarize, compounds with properties that suppress cancer cell growth by inducing cell cycle arrest are highly desired agents in cancer therapy [25]. The aforementioned data suggest that compound **1** weakly induces arrest of the cell cycle, while compounds **4** and **7** are involved mainly in G0/G1 arrest.

### 2.7. GSH:GSSG Ratio

Glutathione (GSH) is an important tripeptide thiol (γ-glutamyl cysteinyl glycine) antioxidant, and its intracellular concentration is an indicator of oxidative stress. Inside the cells, GSH exists in two different forms: a reduced sulfhydryl form (GSH) and glutathione disulfide (GSSG), being an oxidized form [26]. GSH is one of the principal antioxidants involved in many cellular processes, and the GSH: GSSG ratio determines the antioxidant capacity of cells.

Cancer cells contain increased intracellular levels of glutathione (GSH) and activate certain transcription factors, including NF-κB (nuclear factor kappa-light chain-enhancer of activated B cells), HIF (hypoxia-inducible factor 1) and p53 [27]. Cancer cells through the modulation of antioxidative defense systems are able to avoid cell deaths caused by high levels of ROS. Therefore, it is important to evaluate how potential agents influence the intracellular GSH concentration and, subsequently, the GSH: GSSG ratio. In this study, we treated MCF-7 and MDA-MB-231 cells with various concentrations of compounds **1**, **4** and **7** corresponding to ¼ and ½ of their IC_50_ values. Applications of lower concentrations of examined compounds made it possible to observe how they change the GSH: GSSH ratio without significantly affecting cell damage. Results of the effects of selected sulfonamides on intracellular levels of GSH, GSSG and their ratio are presented in Table 6.

In the case of MCF-7 cells, most of the tested compounds did not affect the GSH: GSSG ratio, except for compound **7** at 27.5 µmol/L, which decreased the GSH and GSSG balance (15.9 vs. 21.9 for control). None of the examined sulfonamides influences the GSH: GSSG ratio in MDA-MB-231 cells, which suggests that the antiproliferative properties of these compounds do not result from the effects on the oxidation-reduction balance of cancer cells. Therefore, this activity cannot be considered an additional supporting mechanism in the inhibition of cancer cell growth.

## 3. Materials and Methods 

### 3.1. Cell Culturing

MCF-7 human breast adenocarcinoma cells (HTB-22) were purchased from the American Type Culture Collection (ATCC, Manassas, VA, USA), while MDA-MB-231 human breast adenocarcinoma cells were purchased from Sigma Aldrich (European Collection of Authenticated Cell Cultures (ECACC, Public Health England, Salisbury, UK)).

Both cell lines were cultured in standard conditions (37 °C 5% CO_2_) using Dulbecco’s modified Eagle’s medium (DMEM, Gibco, Thermo Fisher Scientific, Waltham, MA, USA) supplemented with L-glutamine (2 mM, Gibco), heat-inactivated fetal bovine serum (10%, Gibco, Thermo Fisher Scientific, Waltham, MA, USA), penicillin (50 U/mL, Gibco, Thermo Fisher Scientific, Waltham, MA, USA) and streptomycin (50 μg/mL, Gibco, Thermo Fisher Scientific, Waltham, MA, USA). Once the cells reached 80% confluence in culture bottles (75 cm^2^), the cells were washed twice with DPBS solution (Gibco, Thermo Fisher Scientific, Waltham, MA, USA) and harvested using 5 mL of accutase (Sigma Aldrich, St. Louis, MO, USA). For the purpose of the experiments, the cells of passages between 6–10 were used.

### 3.2. Studied Compounds

Sulfonamide derivatives of metformin (compounds **1**–**9**) tested within the paper are presented in Figure 1. The synthesis protocol and basic properties were described previously [24]. The analyzed compounds **1**–**9** are stable in 0.1 M NaOH and TBS buffer.

### 3.3. PMAT and MATE1–2 Gene Expression and Function of OCTs in MCF-7 and MDA-MB-231 Cells

The RNA was extracted by using the E.Z.N.A.® Total RNA Kit I (Omega Bio-tek, Norcross, GA, USA) and converted into cDNA by using M-MuLV reverse transcriptase (400 U), random hexamers (20 µg) and dNTPs (10 mM) (Fermentas, Hanover, MD, USA). Quantification of the MATE1-2 and PMAT genes was performed by employing the Prism 7500 sequence detection system (Applied Biosystems, Inc., Foster City, CA, USA). Briefly, 6 µL of each sample was mixed with 10 µL of PCR reagent mixture (0.5 µL primer probe mix, 5 µL of TaqMan master mix (TaqMan Gene Expression assay, Applied Biosystems, Thermo Fisher Scientific, Waltham, MA, USA) and 0.5 µ sterile water). Transporter gene expression was determined by real-time polymerase chain reaction (RT-PCR) and normalized to endogenous cyclophilin A. The used primer probe mixes included HS00928283 (PMAT and SLC9A4), Hs00217320_m1 (MATE1 and SLC47A1) and Hs00945652_m1 (MATE2 and SLC47A2).

OCT transporters in MCF-7 and MDA-MB-231 cell lines were characterized previously [13]. The studies included characterization of the concentration dependency of choline chloride uptake and time dependency of [^14^C] choline (PerkinElmer, Waltham, MA, USA) uptake. Furthermore, the function of OCT transporters (OCT 1–3) was checked using three known OCT inhibitors: disopyramide, naringin and cimetidine [13].

### 3.4. Inhibition of [^14^C]Choline Uptake

The affinity of sulfonamide derivatives of metformin to OCTs was examined using an OCT substrate, [^14^C] choline. The cells were seeded at a density of 1 × 10^5^ (MCF-7) and 1.25 × 10^5^ (MDA-MB-231) per well on 24-well plates for 24 h. The following day, after removal of the culture medium, the cells were carefully washed with prewarmed HBSS (Hanks’ balanced salt solution) containing 125 mM NaCl, 4.8 mM KCl, 1.2 mM MgSO_4_, 1.2 mM KH_2_PO_4_, 1.3 mM CaCl_2_, 5.6 mM glucose and 25 mM HEPES (pH 7.4). The cells were then preincubated in 500 μL of prewarmed HBSS at 37 °C for 15 min before the addition of pure [^14^C] choline in HBSS (the volume 250 μL) for the uptake experiment (controls) or [^14^C] choline in HBSS and 10−3000 μmol/L of studied compounds (final volume 250 μL). Then, the cells were washed three times with ice-cold HBSS (500 μL) and were lysed with 250 μL of 0.1-M NaOH in the ice bath, and the lysate was mixed with 1.0 mL of an emulsifier safe cocktail (PerkinElmer, Waltham, MA, USA). The radioactivity was measured by liquid scintillation counting (MicroBeta^2^ counter, PerkinElmer, Waltham, MA, USA). The studies were carried out with the use of at least three replicates from the same cell passage.

### 3.5. Uptake Studies

The uptake of metformin sulfonamides (**1**–**9**) in MCF-7 and MDA-MB-231 cells was studied by incubation of the cells with 10−3000 μmol/L of compounds in 250 μL of prewarmed HBSS buffer at 37 °C for 10 min. Subsequently, the cells were then washed three times with 500 μL of ice-cold HBSS and lysed with 250 μL of 0.1-M NaOH. The samples were collected, centrifuged (5 min, 1400 rpm) and the supernatants were analyzed by the HPLC method. The concentration of each compound in the cells was calculated on the basis of the standard curve prepared by spiking known amounts of every compound on the cell layers in 250 μL of 0.1-M NaOH.

The concentration of the studied sulfonamides (**1**–**9**) was assessed using the HPLC system, consisting of an Agilent 1100 binary pump (Agilent Technologies Inc., Wilmington, DE, USA), an 1100 micro-vacuum degasser, an HP 1050 autosampler and an HP 1050 variable wavelength detector (operated at 235 nm). The chromatographic separations of compounds **1**–**9** were conducted on an Agilent Zorbax SB-C18 analytical column (4.6 mm × 150 mm, 5 μm) using an isocratic elution of water containing 0.1% formic acid (pH ca. 3.0) and acetonitrile containing 0.1% formic acid with a changing ratio, depending on the compound. The analyses were conducted at room temperature (RT). The detailed procedure of chromatographic analysis was described in our previous paper [24]. The studies were conducted in quadruplicates.

The protein concentrations in representative wells of each plate were assessed using a Bio-Rad protein assay according to the Bradford method (EnVision, PerkinElmer, Inc., Waltham, MA, USA).

### 3.6. Uptake of Metformin Derivatives in the Presence of OCT and MATE Inhibitors

The uptake of sulfonamide derivatives of metformin (1–9) in MCF-7 and MDA-MB-231 cells was studied also in the presence of disopyramide, cimetidine (OCT1 and 3 inhibitors), lopinavir (OCT and PMAT inhibitor) and methenamine (MATE and OCT2 inhibitors). The cells were incubated (37 °C, 10 min) with compounds 1–9 at concentrations of 400 and 800 μmol/L and inhibitors (disopyramide, cimetidine, lopinavir or methenamine) in 250 μL of prewarmed HBSS buffer. The protocol of determination of the compound concentration was as described above.

### 3.7. Cell Viability and Morphology

The viability of MCF-7 and MDA-MB-231 cells was determined using a colorimetric WST-1 assay (Takara, Takara Bio Europe, Saint-Germain-en-Laye, France). Both cell lines were seeded at a density of 1 × 10^4^ per well on 96-well plates and were cultured for 24 h to obtain 70% confluence. The following day, the cells were treated with various concentrations of compounds **1**–**9** diluted in the cell culture medium (1 and 9). The cells were incubated with tested compounds or pure medium (control) for 24 h (37 °C, 5% CO_2_). Afterwards, the cells were washed with 100 μL of culture medium, and then, the reagent dissolved in the medium was added (100 μL). The plates were incubated at the standard condition for 1.5 h, and the absorbance was read at 450 nm using a microplate reader (iMARK, Bio-Rad, Bio-Rad Laboratories Inc., Hercules, CA, USA). The absorbance recorded for the control samples demonstrated 100% viability. The results of samples treated with the test compounds were expressed as a percentage of the control samples. The data were presented as mean ± standard deviation (SD), *n* = 8. IC_50_ values (the concentration of the tested compound that inhibited cell growth by 50%) were calculated using a concentration-response curve (GraphPad Prism, Software, San Diego, CA, USA).

The effects of compounds **1**–**9** on MCF-7 and MDA-MB-231 cells included also a morphological evaluation. The cells were seeded at a density of 2 × 10^4^ per well on 48-well plates and allowed to obtain 70% confluency. Then, the medium (control) or medium with tested compounds at appropriate concentrations was added. The cells were incubated with compounds for 24 h. After 24 h of cotreatment, the cells were examined using an inverted microscope with phase contrast (Opta-Tech, software OptaView 7, Warsaw, Poland).

### 3.8. Cell Apoptosis Assay

MCF-7 and MDA-MB-231 cells were seeded at a density of 5 × 10^4^ per well on 24-well plates and incubated for 24 h (37 °C, 5% CO_2_) to obtain 70 % confluency. Then, the medium (control) or medium with the tested compounds in a volume of 250 μL was added and incubated for another 24 h. The cells were harvested using accutase (Sigma Aldrich, St. Louis, MO, USA) as a detaching reagent, collected to Eppendorf tubes and centrifuged (1100 rpm, 5 min). Afterwards, the cells were resuspended in 1 mL of a cold cell-staining buffer (Biolegend, London UK) and washed twice with a cell-staining buffer. The final step included suspension of the cell pellets in 100 μL of a binding buffer, staining with propidine iodide (PI; 10 μL) and FITC-annexin solutions (AV; 5 μL) (FITC Annexin V Apoptosis Detection Kit with PI (Biolegend, UK)). The samples were incubated for 20 min at room temperature in the dark, followed by an analysis conducted on a cytometer (CytoFlex, blue laser, 480 nm, Beckman-Coulter, Indianapolis, IN, USA). The results were analyzed using Kaluza 2.1 (Beckman-Coulter) software.

Annexin V (−) and PI (−) cells were considered living cells, annexin V (+) and PI (−) as early-apoptotic cells, annexin V (+) and PI (+) as late-apoptotic cells and annexin V (−) and PI (+) as necrotic cells.

The experiments were conducted in triplicates (*n* = 3). The coefficients of variation for the assay were determined (CV = 0.97–13.62%, depending on the calculated cell population).

### 3.9. Cell Cycle Analysis

MCF-7 and MDA-MB-231 cells were seeded in 6-well plates at a density of 2 × 10^5^ cells per well and cultured for 24 h under standard conditions (with 2 mL of the medium). Next, cells were exposed to the tested compounds at concentrations corresponding to IC_50_. After 24 h of treatment, control and treated cells were then collected (washed with cold PBS and treated with accutase), transferred to Eppendorf tubes and centrifuged (1100 rpm, 5 min, 5 °C). The supernatant was discarded, cold PBS was added (1 mL) and the cells were centrifuged (1200 rpm, 5 min). The solution was removed, and the cells were fixed in cold 70% ethanol at 4 ºC. Prior to the analysis, the cells were centrifuged (3600 rpm, 5 min) and washed twice with PBS. The solution was discarded, the pellets were suspended in 200 µL of PBS and incubated with ribonuclease at 37 ºC (25 µL, final concentration in a sample was 100 µg/mL) for 30 min and stained with propidine iodide (12.5 µL, final concentration in a sample was 50 µg/mL) in darkness at room temperature for additional 20 min. The final volume of the sample was 250 µL.

DNA content and the number of cells in individual cell cycle phases was measured by flow cytometry (CytoFlex, blue laser, 480 nm, Beckman-Coulter, Indianapolis, IN, USA). Cells (20,000) were analyzed from each sample. The percentage of cells in each cell cycle phase was calculated from live cells that were expressed as 100%. The data are presented as the mean ± SD of three independent experiments. The coefficients of variation for the assay were established (CV = 0.37–11.78%, depending on the cell population).

### 3.10. GSH:GSSG Ratio

The quantitative determination of glutathione and glutathione disulfide was performed using the enzymatic recycling method according to the protocol of Rahman [26]. The cells were seeded at a density of 2 × 10^5^ per well in a 6-well plate and cultured for 48 h. Then, the cells were treated with tested compounds 1, 4 and 7 at their respective ¼ and ½ IC_50_ values for MCF-7 and MDA-MB-231 cells. Additionally, controls with pure medium, AAPH (2,2′-Azobis(2-methylpropionamidine) dihydro-chloride, Sigma Aldrich, St. Louis, MO, USA) and ascorbic acid (Polish Chemical Reagents, Poland) were performed. The cells were incubated with compounds for another 24 h, then washed twice with 2 mL of cold, Ca^2+^-/Mg^2+^-free PBS (Biomed Lublin, Lublin, Poland). The cells were harvested using 1-mL accutase (Sigma Aldrich, St. Louis, MO, USA), and the wells were washed with 1 mL of cold growth medium. The cells were transferred to prechilled 2.0-mL Eppendorf tubes and centrifuged at 1000× *g* for 5 min at 4 °C. The supernatant was discarded, the pellets were resuspended in 1 mL of cold PBS, the number of cells was determined in each sample and the cellular suspension was centrifuged again. PBS was discarded, and the pellets were stored covered with 30-µL PBS at –70 °C. The following day, the cells were suspended in 0.5 mL of ice-cold extraction buffer (0.1% Triton-X (Polish Chemical Reagents, Gliwice, Poland) and 0.6% sulfosalicylic acid (Sigma Aldrich) in a potassium phosphate buffer with EDTA (Sigma Aldrich)) and sonicated in icy water for 3 min with vortexing every 30 s. The cells were frozen once again at −80 °C, defrosted on ice and frozen once again. Following the second freeze–thaw cycle, the cells were centrifuged at 1500× *g* for 5 min at 4 °C, and then, immediately, the supernatant was transferred to prechilled Eppendorf tubes. The samples were divided into two parts: one was used to measure GSH, and the other one was derivatized by an addition of 4 µL of 2-vinylpyridine (diluted 1:10 with phosphate buffer) (Sigma Aldrich, St. Louis, MO, USA) to determine the GSSG content.

GSH measurements included an addition of 20 µL of the tested sample, blank or GSH standard, 120 µL of the mixture of an equal volume of DTNB solution (2 mg dissolved in 3 mL of phosphate buffer, Sigma Aldrich) and glutathione reductase (10 U in 3 mL of phosphate buffer, Sigma Aldrich, St. Louis, MO, USA) and 30 s of incubation to allow the conversion of GSSG to GSH. The final step included the addition of 60 µL of β-NADPH (2 mg dissolved in 3 mL of phosphate buffer, Sigma Aldrich). The absorbance was read immediately at 415 nm in a microplate reader (Bio-Rad), and the measurements were taken every 30 s for 2 min (6 readings in total). The amount of GSH was calculated on the basis of the calibration curve prepared using GSH (Sigma Aldrich, St. Louis, MO, USA) at concentrations ranging from 0.040 to 2.6 nmol/mL.

The quantification of GSSG in the test samples was performed according to the same procedure as GSH. The samples treated with 2-VP were incubated for one hour at room temperature, and then, 20 µL of the tested sample was analyzed using the same reagents which were applied in the GSH measurements. The amount of GSSG was calculated on the basis of the calibration curve, prepared using GSSG (Sigma Aldrich) at concentrations of 0.040–2.6 nmol/mL. The results are presented as mean ± SD, *n* = 6–8 and expressed as nmol per 1 mL of cells.

### 3.11. Data Analysis

The statistical calculations were performed using a commercially available package (Statistica 12.0, StatSoft; GraphPad Prism 5, San Diego, CA, USA). The results are presented as the mean ± standard deviation (SD) for variables with a normal distribution of values. The normal distribution of continuous variables was verified with the Shapiro-Wilk test, while homogeneity of the variances was checked using the Levene test. Statistical differences between groups were tested using one-way ANOVA, followed by a subsequent post hoc test (Dunnett’s or Tukey’s test). The variables with non-normal distributions were compared using the Wilcoxon signed rank test. The results of all the tests were considered significant at *p*-values lower than 0.05.

The concentrations contributing to the 50% of cell growth inhibition (IC_50_) were calculated using nonlinear regression analysis (fitting the curve to log (concentration) vs. cellular response).

## 4. Conclusions

To conclude, in this study, we evaluated how chemical modifications of the metformin scaffold into sulfonamides with a halogenated aromatic ring may affect their affinity towards OCTs, their cellular uptake and antiproliferative efficacy. Nine sulfonamide metformin derivatives with Cl, Br or F substituents in the benzene ring (compounds **1**–**9**) were examined in the MCF-7 and MDA-MB-231 cell lines. The uptake of most sulfonamides was more efficient in MCF-7 cells than in MDA-MB-231 cells. Considering the type of halogen substituents, it can be concluded that the presence of a chlorine atom in the aromatic ring contributes to the largest uptake in MCF-7 cells. Based on the obtained results, we suppose that the considerably lower uptake of **1**–**3** in MDA-MB-231 cells results from a weak affinity towards the transporters and their lower capacity. The studies with transporter inhibitors suggest that chlorine derivatives use OCT and PMAT transporters in MCF-7 cells. Interestingly, both *p*-chlorine and *p*-bromine derivatives (compounds **3** and **6**) were characterized by relatively good uptakes in both cell lines. In addition, compounds **3** and **6** were found to interact not only with OCT transporters but, also, with PMAT and MATE1. Derivatives with a fluorine substituent in the benzene ring were also characterized by greater uptakes in MCF-7 cells than in MDA-MB-231 cells.

The increased uptakes of sulfonamide derivatives of metformin in MCF-7 were accompanied by the promising antiproliferative potency of studied compounds, with compound **1** being the most active (IC_50_ = 12.6 ± 1.2 µmol/L). These results indicate that chemical modification of the metformin backbone into halogenated sulfonamides leads to obtaining compounds with much better cellular uptakes in MCF-7 cells and subsequent higher cytotoxic properties than the parent drug, metformin, which, as it was previously found, did not significantly affect MCF-7 and MDA-MB-231 growth up to 3000 µmol/L. Cytotoxic properties of compounds **1**, **4** and **7** were associated with the induction of early and late apoptosis in MCF-7 cells. Furthermore, compounds **4** and **7** were found to significantly arrest the cell cycle at the G0/G1 phase.

Collectively, this is the first study that provides evidence that the chemical modification of the biguanide backbone into halogenated sulfonamides leads to improved transporter-mediated cellular uptakes in MCF-7 and affects the antiproliferative potency of studied compounds through the inductions of apoptosis and cell cycle arrest.

## Figures and Tables

**Table 1:** The effects of sulfonamide derivatives of metformin on [^14^C] choline uptake in MCF-7 and MDA-MB-231 cells. The results (IC_50_ values, µmol/L) are presented as mean ± SD (*n* = 3).

Compound	IC_50_ MCF-7 (μmol/L)	IC_50_ MDA-MB-231 (μmol/L)
1	1053 ± 14.2	2135 ± 14.0
2	2923 ± 9.1	4449 ± 18.1
3	NE	3757.1 ± 124.4
4	1670 ± 8.5	919.60 ± 13.0
5	888.5 ± 11.4	593.1 ± 15.0
6	2062.5 ± 58.4	2489.9 ± 211.1
7	6354 ± 12.9	5149 ± 22.0
8	7195 ± 5.3	2284 ± 14.0
9	1057 ± 7.5	1383 ± 14.0

NE—not estimated up to 46% at the concentration of 2000 μmol/L (logarytmic phase).

**Table 2:** Eadie-Hofstee analysis of the uptake of sulfonamide derivatives of metformin in MCF-7 and MDA-MB-231 cells (Km and Vmax values).

		Kinetic Parameters of Prodrug Uptakes			
		MCF-7		MDA-MB-231 Cells	
Compound	Transp./Efficacy (mL/(min∙mg))	Km (μmol/L)	Vmax (nmol/min/mg)	Km (μmol/L)	Vmax (nmol/min/mg)
**1**	I	6719.0 ± 981.9	18.53 ± 2.41	3070.0 ± 561.6	0.0182 ± 0.0026
	Vmax/Km ^#^	0.00276		0.000006	
	II	21130.0 ± 7726	62.84 ± 21.37	3298.0 ± 625.0	0.0261 ± 0.0040
	Vmax/Km ^#^	0.00297		0.000008	
**2**	I	4014.0 ± 695.8	19.2 ± 2.31	2404.0 ± 353.3	0.077 ± 0.008
	Vmax/Km ^#^	0.00478		0.00003	
	II	7474.0 ± 1821.0	24.91 ± 5.44	5475.0 ± 730.0	0.111 ± 0.016
	Vmax/Km ^#^	0.00333		0.00002	
**3**	I	812.7 ± 86.50	1.614 ± 0.11	2366.0 ± 479.9	1.390 ± 0.23
	Vmax/Km ^#^	0.0019		0.0006	
	II	8814 ± 791.3	10.76 ± 0.88	4666.0 ± 932.4	2.20 ± 0.41
	Vmax/Km ^#^	0.0012		0.0005	
**4**	I	1540.0 ± 220.3	0.031 ± 0.002	5516.0 ± 748.2	10.27 ± 1.18
	Vmax/Km ^#^	0.0000201		0.0018	
	II	8367.0 ± 1425.0	0.153 ± 0.022	11570.0 ± 2436	21.44 ± 4.03
	Vmax/Km ^#^	0.0000183		0.0018	
**5**	I	3616.0 ± 619.2	1.635 ± 0.21	467.7 ± 108.8	0.038 ± 0.004
	Vmax/Km ^#^	0.00045		0.000081	
	II	6846.0 ± 712.9	2.167 ± 0.196	1071 ± 204.0	0.098 ± 0.011
	Vmax/Km ^#^	0.000316		0.000092	
**6**	I	307.6 ± 101.8	0.7898 ± 0.12	1934.0 ± 222.8	2.644 ± 0.22
	Vmax/Km ^#^	0.00257		0.00137	
	II	8933.0 ± 1099	13.64 ± 1.55	2110.0 ± 172.4	2.091 ± 0.15
	Vmax/Km ^#^	0.00153		0.00099	
**7**	I	2134.0 ± 814.5	5.303 ± 1.334	1535.0 ± 298.4	0.262 ± 0.035
	Vmax/Km ^#^	0.00248		0.00017	
	II	6141.0 ± 4603	22.43 ± 12.78	2250.0 ± 189.7	0.443 ± 0.026
	Vmax/Km ^#^	0.00365		0.0002	
**8**	I	2837.0 ± 778.2	0.830 ± 0.171	1235.0 ± 1313.0	0.049 ± 0.025
	Vmax/Km ^#^	0.000292		0.00004	
	II	NE	NE	3383.0 ± 2133	0.045 ± 0.021
	Vmax/Km ^#^	0.00194		0.000013	
**9**	I	NE	NE	1880.0 ± 428.6	0.097 ± 0.015
	Vmax/Km ^#^	NE		0.00002	
	II	NE	NE	2219.0 ± 617.7	0.053 ± 0.012
	Vmax/Km ^#^	NE		0.000024	

^#^ The unit of Vmax/Km (mL/(min∙mg∙protein)); NE—not established.

**Table 3:** The effects of metformin derivatives on the inhibition of MCF-7 and MDA-MB-231 cells’ viability. The results (IC_50_ values, µmol/L) are presented as mean ± SD (*n* = 6–8).

COMPOUND	MCF-7 Cells (µmol/L)	MDA-MB-231 Cells (µmol/L)
**1**	12.6 ± 1.2	69.25% ^#^
**2**	533.2 ± 87.4	843.9 ± 135.0
**3**	1430 ± 135.0	1042 ± 134.0
**4**	429.7 ± 119.0	56.37% ^#^
**5**	106.2 ± 12.3	49.81% ^#^
**6**	1142 ± 139.0	2106 ± 137.0
**7**	55.05 ± 2.74	2594 ± 150
**8**	171.1 ± 13.6	67.12% ^#^
**9**	421.6 ± 32.8	70.91% ^#^

^#^ The percentage of viability at 3000 µmol/L.

**Table 4:** Annexin V-FITC/PI double-staining analysis of apoptosis in MCF-7 and MDA-MB-231 cells.

Compound [μmol/L]	Single Cells in Gate B [%] ^1^	Living Cells [E−−] [%] ^2^	Necrotic Cells [E−+] [%] ^2^	Early Apoptotic [E+−] [%] ^2^	Late Apoptotic [E++] [%] ^2^
Control MCF-7	87.90 ± 0.87	88.59 ± 0.96	0.93 ± 0.33	5.84 ± 0.80	4.64 ± 0.57
Comp. 1 [6.5 μmol/L]	77.51 ± 0.92 ***	32.82 ± 4.50 ***	0.67 ± 0.08	27.59 ± 1.03 ***	38.93 ± 3.46 ***
Comp. 1 [13 μmol/L]	82.66 ± 0.68 ***	29.96 ± 6.41 ***	0.41 ± 0.02 **	35.79 ± 3.78 ***	33.84 ± 3.17 ***
Comp. 4 [215 μmol/L]	78.90 ± 0.11 ***	34.17 ± 3.88 ***	0.45 ± 0.15 **	30.69 ± 3.01 ***	34.69 ± 1.51 ***
Comp. 4 [430 μmol/L]	83.19 ± 0.40 **	16.84 ± 1.93 ***	0.29 ± 0.09 ***	22.71 ± 2.11 ***	60.16 ± 2.21 ***
Comp. 7 [27.5 μmol/L]	78.97 ± 0.51 ***	39.85 ± 5.67 ***	0.57 ± 0.03 *	23.59 ± 1.57 ***	35.98 ± 4.64 ***
Comp. 7 [55 μmol/mL]	79.48 ± 0.82***	31.88 ± 4.77 ***	0.54 ± 0.01 *	24.35 ± 2.19 ***	43.24 ± 4.09 ***
Control MDA-MB-231	93.49 ± 0.47	90.68 ± 1.37	2.27 ± 0.33	3.75 ± 1.39	3.31 ± 0.32
Comp. 1 [1500 μmol/L]	94.34 ± 0.26	81.55 ± 1.65 *	2.74 ± 0.98	7.66 ± 0.06 ***	8.06 ± 0.81 ***
Comp. 1 [3000 μmol/L]	91.85 ± 0.37 **	53.83 ± 3.87 ***	4.81 ± 0.60	9.96 ± 0.92 ***	31.41 ± 3.46 ***
Comp. 4 [1500 μmol/L]	93.37 ± 0.34	72.64 ± 1.76 ***	6.31 ± 0.26 *	7.74 ± 0.91 ***	13.30 ± 0.66 ***
Comp. 4 [3000 μmol/L]	90.59 ± 0.70 ***	49.49 ± 2.31 ***	7.15 ± 1.45 **	6.85 ± 0.34 **	36.51 ± 3.36 ***
Comp. 7 [1300 μmol/L]	93.75 ± 0.41	68.26 ± 3.51 ***	4.06 ± 1.75	8.78 ± 0.63 ***	18.89 ± 2.67 ***
Comp. 7 [2600 μmol/mL]	91.60 ± 0.48 **	50.40 ± 4.95 ***	7.66 ± 2.93 **	6.39 ± 0.16 **	35.54 ± 2.67 ***

MCF-7 cells were treated with tested compounds **1**, **4** and **7** at concentrations corresponding to ½ × IC_50_ and IC_50_ values for 24 h followed by staining with annexin V FITC and propidium iodide (PI). MDA-MB-231 cells were treated with compounds **1** and **4** at 1500 and 3000 µmol/L and compound **7** at concentrations corresponding to ½ × IC_50_ and IC_50_ values for 24 h, followed by staining with annexin V FITC and propidium iodide (PI) ^1^—single cells gathered within gate B reflecting the % of the absolute number of acquired events and ^2^—the single cells gathered in gate B were divided depending on staining with annexin V and PI: (E−−)—living cells, (E−+)—necrotic cells, (E+−)—early-apoptotic cells and (E++)—late-apoptotic cells. The results are presented as mean ± standard deviation (SD), *n* = 3. The statistically significant differences between samples and respective controls are depicted by * (* *p* < 0.05; ** *p* < 0.01 and *** *p* < 0.001).

**Table 5:** The effects of selected sulfonamides on cell cycle progression in MCF-7 and MDA-MB-231 cells.

Compound [µmol/L]	Sub G0/G1 [%]	G0/G1 [%]	S [%]	G2/M [%]
Control MCF-7	1.65 ± 0.19	45.56 ± 0.68	33.63 ± 0.02	19.20 ± 0.90
Comp. 1 [13 µmol/L]	8.28 ± 1.04 ***	38.76 ± 0.80 **	35.45 ± 0.23 **	17.54 ± 0.48 *
Comp. 4 [430 µmol/L]	2.98 ± 0.40	73.69 ± 0.26 ***	15.16 ± 0.54 ***	8.21 ± 0.03 ***
Comp. 7 [55 µmol/L]	17.85 ± 2.08 ***	56.48 ± 3.34 ***	10.34 ± 0.74 ***	15.43 ± 0.93 ***
Control MDA-MB-231	1.96 ± 0.11	50.54 ± 0.86	28.88 ± 0.45	18.50 ± 0.45
Comp. 7 [2600 µmol/L]	3.41 ± 0.08 **	64.31 ± 1.13 ***	19.99 ± 0.97 **	12.24 ± 0.10 **

MCF-7 cells were treated with tested compounds **1**, **4**, **7** at concentrations corresponding to IC_50_ values for 24 h followed by staining with propidium iodide (PI). MDA-MB-231 cells were treated with compound **7** at a concentration of 2600 µmol/L (IC_50_ value). DNA content was determined using a flow cytometry analysis of the PI-stained cells. The results are presented as mean ± standard deviation (SD), *n* = 3. The statistically significant differences between samples and respective controls are depicted by * (* *p* < 0.05, ** *p* < 0.01 and *** *p* < 0.001).

**Table 6:** The effects of sulfonamides **1**, **4** and **7** on the intracellular GSH and GSSG concentrations.

	MCF-7			MDA-MB-231		
	GSH [nmol/1 mln Cells]	GSSG [nmol/1 mln Cells]	GSH:GSSG Ratio	GSH [nmol/1 mln Cells]	GSSG [nmol/1 mln Cells]	GSH:GSSG Ratio
CTR	147.1 ± 9.6	7.0 ± 0.9	21.9	204.3 ± 21.0	17.9 ± 1.3	11.4
Ascorbic acid [200 µM]	153.1 ± 5.4	6.0 ± 0.4	25.3	249.6 ± 20.8	17.9 ± 1.5	13.9
AAPH [10 mM]	117.6 ± 7.8	12.8 ± 0.5	8.9	102.7 ± 3.2	14.3 ± 1.9	6.9
Comp. 1 [1/2 × IC_50_]	167.5 ± 5.1	7.4 ± 1.8	22.6	242.0 ± 47.0	19.9 ± 2.0	12.1
Comp. 1 [1/4 × IC_50_]	183.1 ± 8.9	9.1 ± 3.9	20.1	238.0 ± 4.3	19.5 ± 2.2	12.2
Comp. 4 [1/2 × IC_50_]	148.5 ± 4.8	6.2 ± 0.7	24.0	242.1 ± 13.7	19.6 ± 1.4	12.3
Comp. 4 [1/4 × IC_50_]	165.4 ± 9.6	8.6 ± 0.7	19.2	250.2 ± 12.1	19.9 ± 2.3	12.5
Comp. 7 [1/2 × IC_50_]	169.0 ± 6.0	10.6 ± 0.4	15.9	248.7 ± 13.3	21.9 ± 2.9	11.3
Comp. 7 [1/4 × IC_50_]	166.8 ± 12.8	7.9 ± 0.9	21.2	265.0 ± 15.9	22.5 ± 0.9	11.8

IC_50_ values in MCF-7 and MDA-MB-231 cells are presented in Table 3. The cells were incubated with tested compounds for 24 h. Ascorbic acid and 2,2′-Azobis(2-methylpropionamidine) dihydrochloride (AAPH) were used as protective and ROS-inducer agents, respectively. GSH and GSSG concentrations in MCF-7 and MDA-MB-231 cells were determined using a spectrophotometric method with Ellman’s reagent. The results are expressed as mean ± SD, *n* = 6–8.

## Supplementary Materials

Supplementary materials can be found at https://www.mdpi.com/1422-0067/21/7/2389/s1. Table S1. Km and vmax values for the uptake of sulfonamide derivatives 1–9 in MCF-7 and MDA-MB-231 cells (Michaelis Menten curves); Table S2. The summary of interactions of metformin derivatives 1–9 with OCT, PMAT and MATE1 transporters in MCF-7 and MDA-MB-231 cells. Figure S1. The expression of transporters in MCF-7 and MDA-MB-231 cells: A) MATE 1 – 2, and PMAT transporters; B) OCT 1–3. Figure S2. The uptake mechanism of compounds 4 - 6 (400 and 800 µmol/L) into MCF-7 cells and MDA-MB-231 cells. Figure S3. The uptake of selected sulfonamides into MCF-7 or MDA-MB-231 cells. Figure S4. The uptake mechanism of compounds 7–9 (400 and 800 µmol/L) into MCF-7 cells and MDA-MB-231 cells.
