# Supplementary material for: Effective Cellular Transport of Ortho-Halogenated Sulfonamide Derivatives of Metformin Is Related to Improved Antiproliferative Activity and Apoptosis Induction in MCF-7 Cells
Source: Int J Mol Sci. 2020 Mar 30;21(7):2389. doi: 10.3390/ijms21072389 (PMC7177633; doi:10.3390/ijms21072389)
Supplement: Supplementary file 1 [file ijms-21-02389-s001.pdf]

Table S1. Km and vmax values for the uptake of sulfonamide derivatives **1–9** in MCF-7 and MDA-MB-231 cells (Michaelis Menten curves).

	Kinetic parameters of sulfonamide uptake					
	MCF-7			MDA-231 cells		
COMPOUND	**Km** [μmol/L]	**Vmax** [nmol/min/mg]	**Vmax/Km** [mL/(nim*mg)]	**Km** [μmol/L]	**Vmax** [nmol/min/mg]	**Vmax/Km** [mL/(nim*mg)]
**Metformin**	5583 ± 1560#	0.801 ± 0.296#	0.00014#	3375.0 ± 952#	0.718 ± 0.181#	0.000212#
**1**	NE	NE	NE	8300 ± 3266	0.047 ± 0.065	0.000006
**2**	9051 ± 1521	34.4 ± 17.2	0.0038	6752 ± 1731	0.157 ± 0.129	0.000023
**3**	3584 ± 1711	5.073 ± 1.96	0.00141	NE	NE	NE
**4**	2471 ± 652	0.049 ± 0.02	0.00002	NE	NE	NE
**5**	4938 ± 1342	1.87 ± 0.95	0.00038	NE	NE	NE
**6**	NE	NE	NE	3753.0 ± 1604	4.044 ± 1.302	0.00107
**7**	10768 ± 5128	23.92 ± 7.20	0.0022	NE	NE	NE
**8**	NE	NE	NE	7402 ± 510	0.126 ± 0.032	0.000017
**9**	NE	NE	NE	NE	NE	NE

NE – not estimated (linear dependency up to the maximal tested concentrations); # kinetic parameters of metformin uptake in MCF-7 cells and MDA-MB-231 cells were reported previously (Markowicz-Piasecka et al., 2019).

Table 2. The summary of interactions of metformin derivatives 1–9 with OCT, PMAT and MATE1 transporters in MCF-7 and MDA-MB-231 cells.

**COMPOUND**	**MCF-7**	**MDA-MB-231**
**1**	OCT1, OCT3, PMAT, MATE1	OCT1, PMAT
**2**	OCT1, OCT3, PMAT	OCT1, OCT3
**3**	OCT1,MATE1	OCT1, PMAT, MATE1
**4**	OCT1, PMAT	OCT1, OCT3, PMAT
**5**	OCT1, MATE1, PMAT	OCT1, OCT3
**6**	OCT1, PMAT, MATE1	OCT1, OCT3, MATE1
**7**	OCT3, PMAT	OCT1, OCT3
**8**	OCT1	OCT1, OCT3
**9**	PMAT	OCT1, OCT3

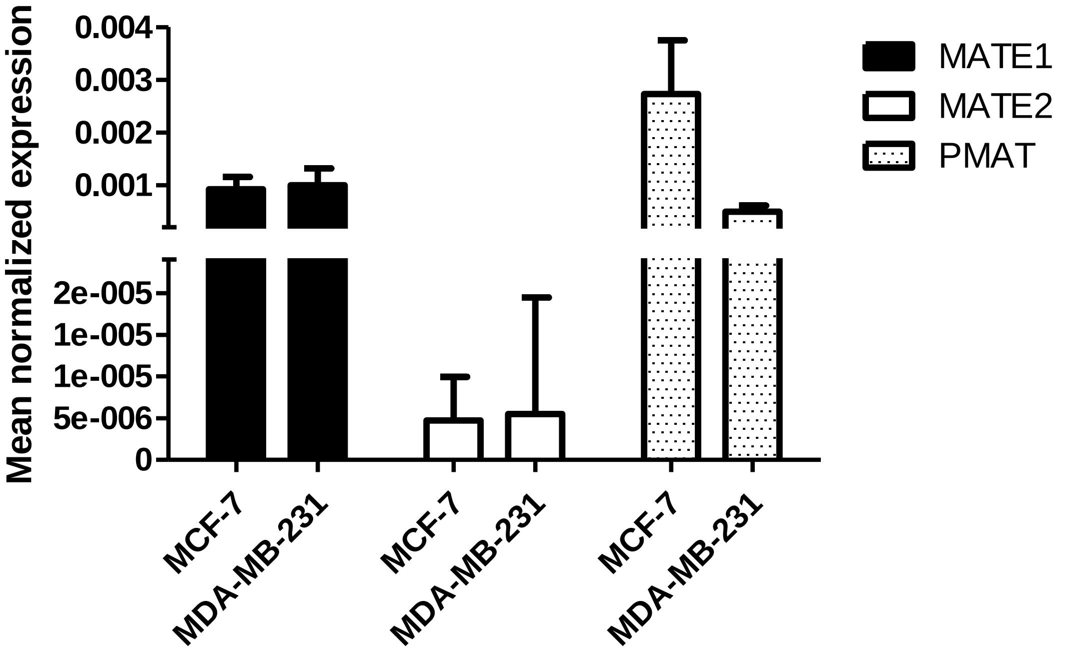

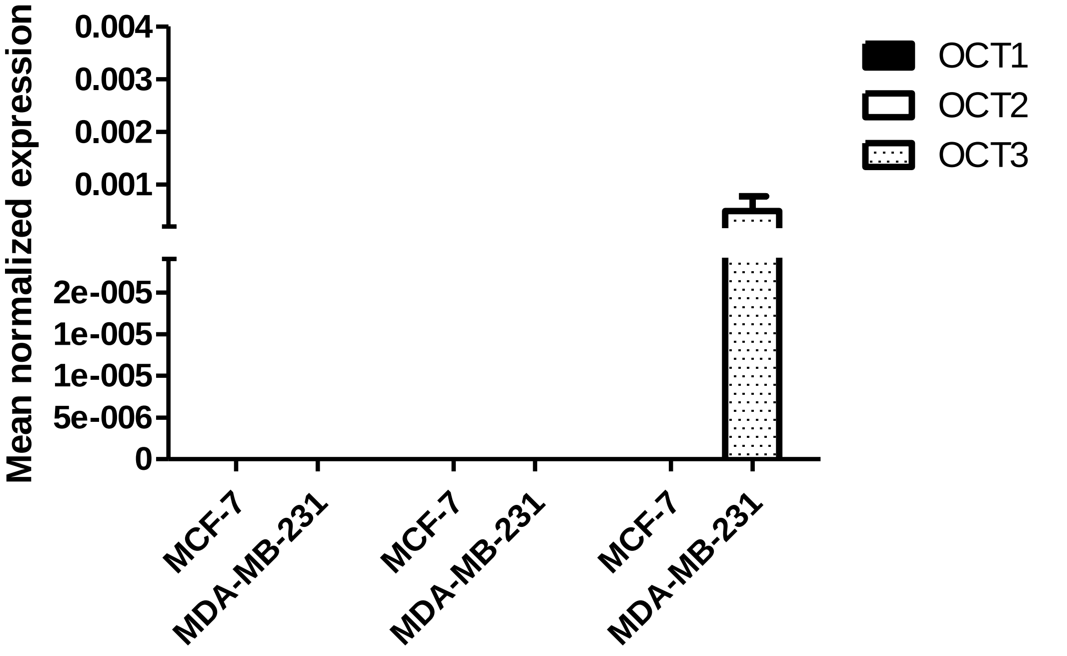

Figure S1. The expression of transporters in MCF-7 and MDA-MB-231 cells: A) MATE 1 – 2, and PMAT transporters; B) OCT 1 – 3. The results of OCT1–3 expression were published previously (Markowicz-Piasecka et al., 2019).

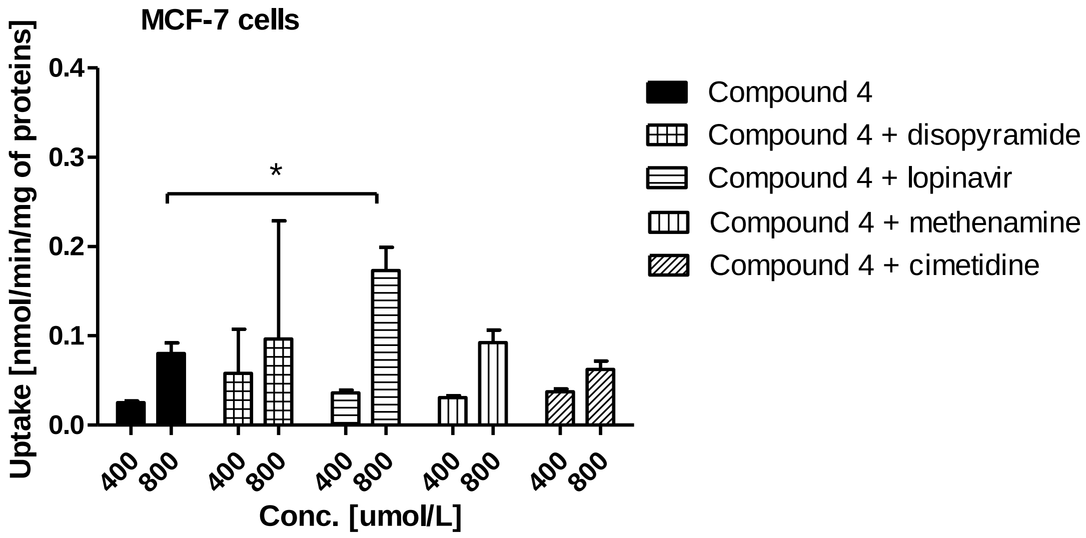
MCF-7 cells
Uptake [nmol/min/mg of proteins]
0.0
0.1
0.2
0.3
0.4
*
400
800
Conc. [umol/L]
Compound 4
Compound 4 + disopyramide
Compound 4 + lopinavir
Compound 4 + methenamine
Compound 4 + cimetidine

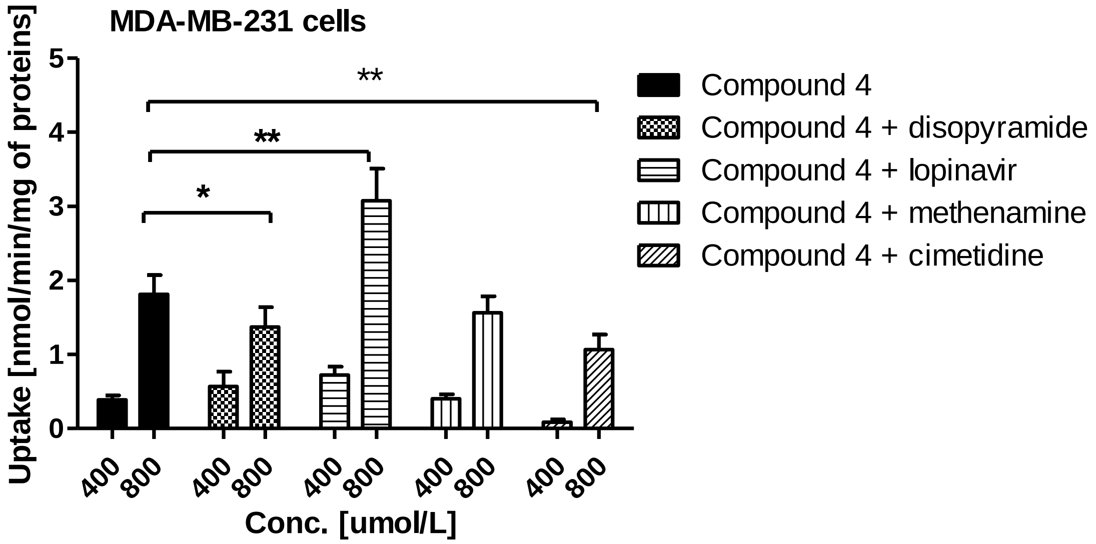
MDA-MB-231 cells
Uptake [nmol/min/mg of proteins]
0
1
2
3
4
5
*
**
**
400
800
Conc. [umol/L]
Compound 4
Compound 4 + disopyramide
Compound 4 + lopinavir
Compound 4 + methenamine
Compound 4 + cimetidine

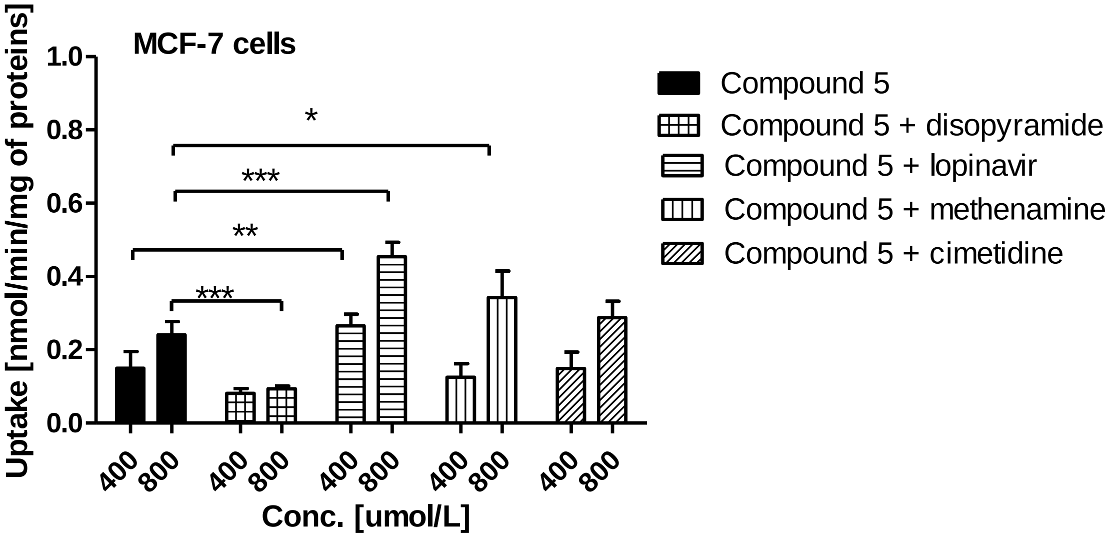
MCF-7 cells
Uptake [nmol/min/mg of proteins]
1.0
0.8
0.6
0.4
0.2
0.0
*
***
**
***
400
800
Conc. [umol/L]
Compound 5
Compound 5 + disopyramide
Compound 5 + lopinavir
Compound 5 + methenamine
Compound 5 + cimetidine

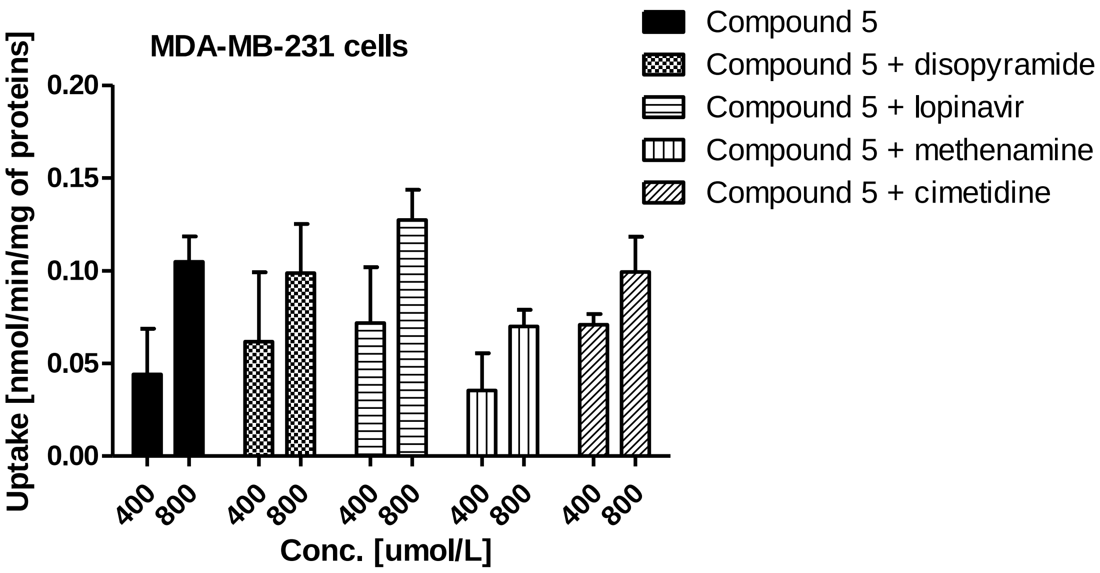
MDA-MB-231 cells
Uptake [nmol/min/mg of proteins]
0.20
0.15
0.10
0.05
0.00
400
800
Conc. [umol/L]
Compound 5
Compound 5 + disopyramide
Compound 5 + lopinavir
Compound 5 + methenamine
Compound 5 + cimetidine

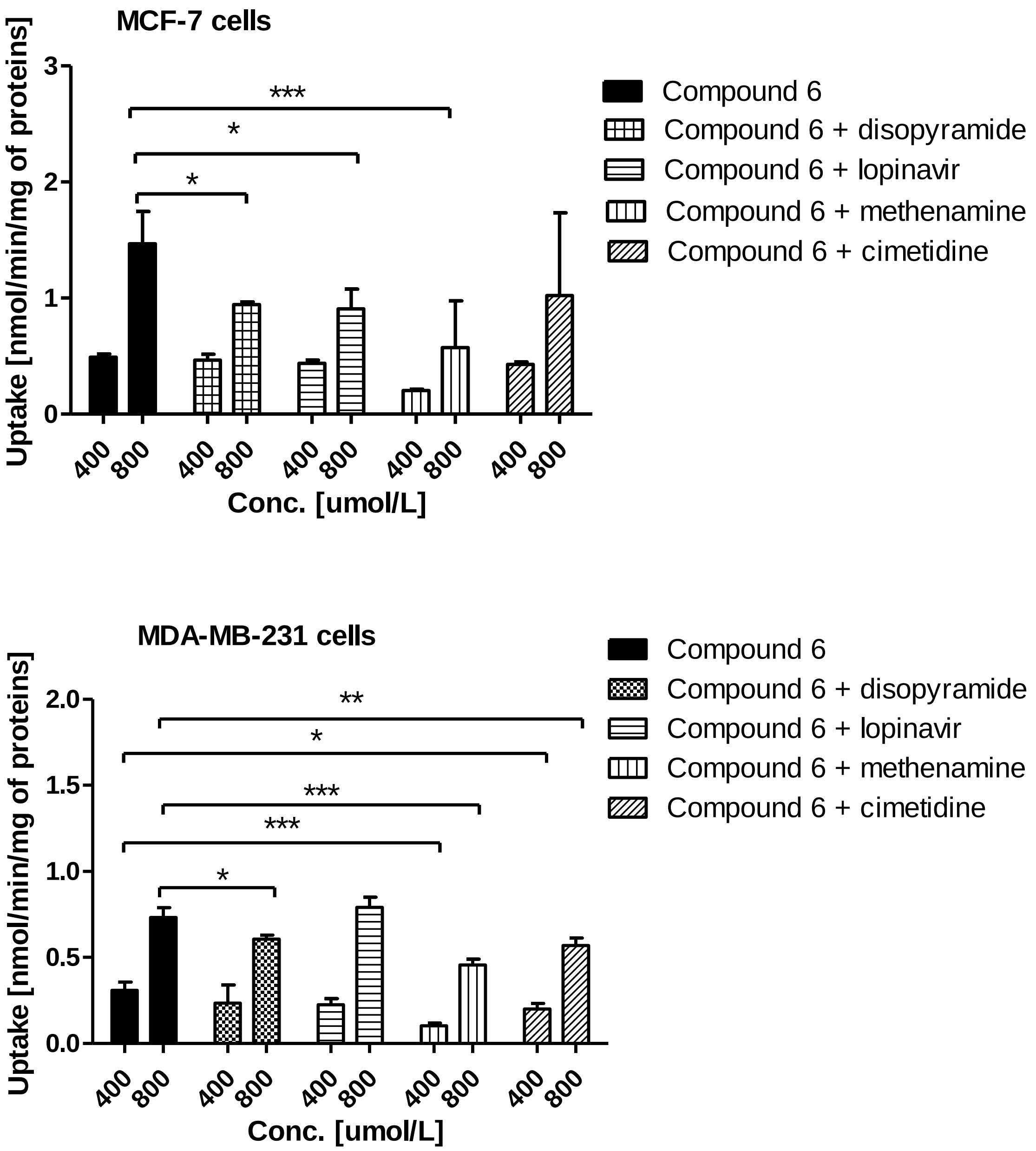

Figure S2. The uptake mechanism of compounds **4–6** (400 and 800 μmol/L) into MCF-7 cells and MDA-MB-231 cells. The uptake was determined in the presence of OCT and MATE inhibitors, disopyramide, lopinavir, methenamine, cimetidine (400 and 800 μmol/L) for 10 minutes at 37 °C. One-way Anova analysis was performed to compare the uptake of pure compounds (**4–6** at 400 and 800 μmol/L) with their uptake in the presence of transporters inhibitors. The significant differences between the uptake of pure compounds **4–6** (black bars) and their respective mixtures with inhibitors (disopyramide, lopinavir, methenamine or cimetidine) are marked with black lines and are denoted with asterisk. * $p < 0.05$, ** $p < 0.01$, *** $p < 0.001$.

A)

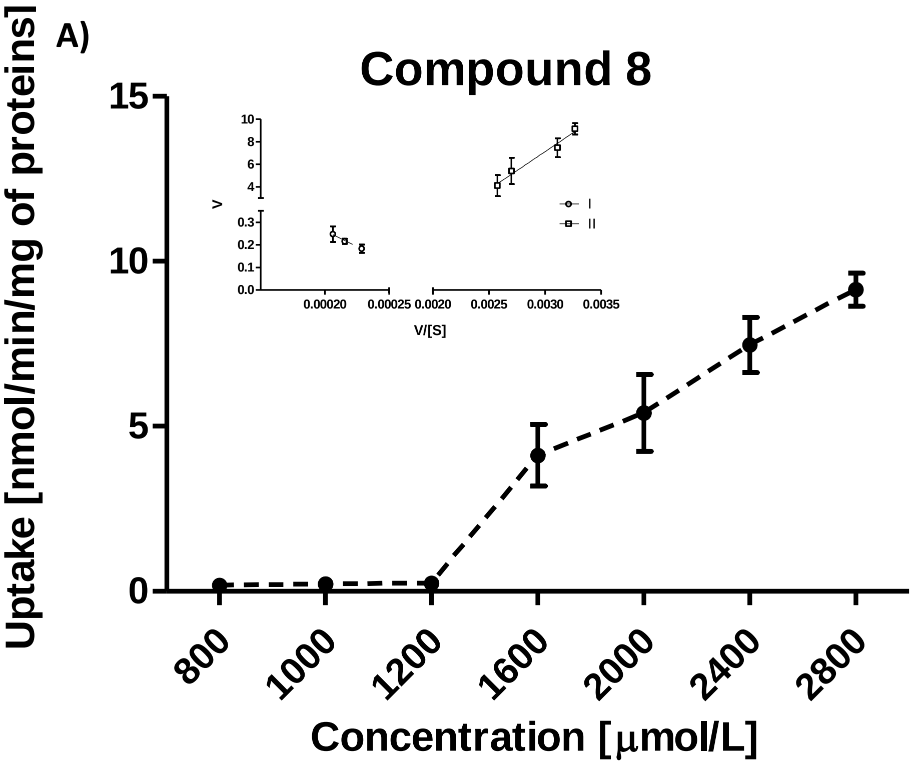
Compound 8
Uptake [nmol/min/mg of proteins]
15
10
5
0
800
1000
1200
1600
2000
2400
2800
Concentration [µmol/L]
V
V/[S]
I
II

B)

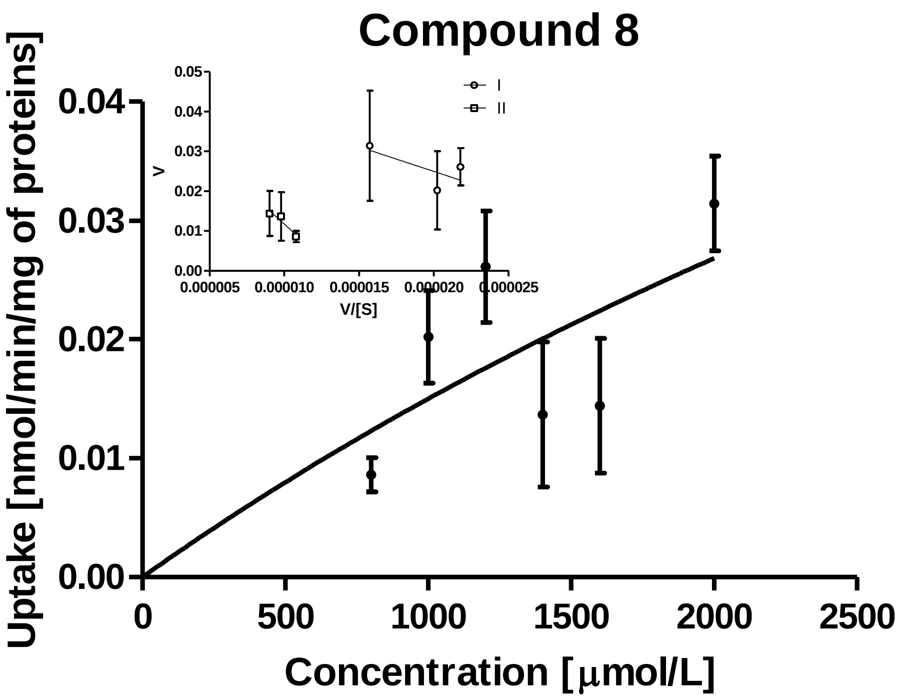
Compound 8
Uptake [nmol/min/mg of proteins]
0.04
0.03
0.02
0.01
0.00
0
500
1000
1500
2000
2500
Concentration [µmol/L]
V
V/[S]
I
II

C)

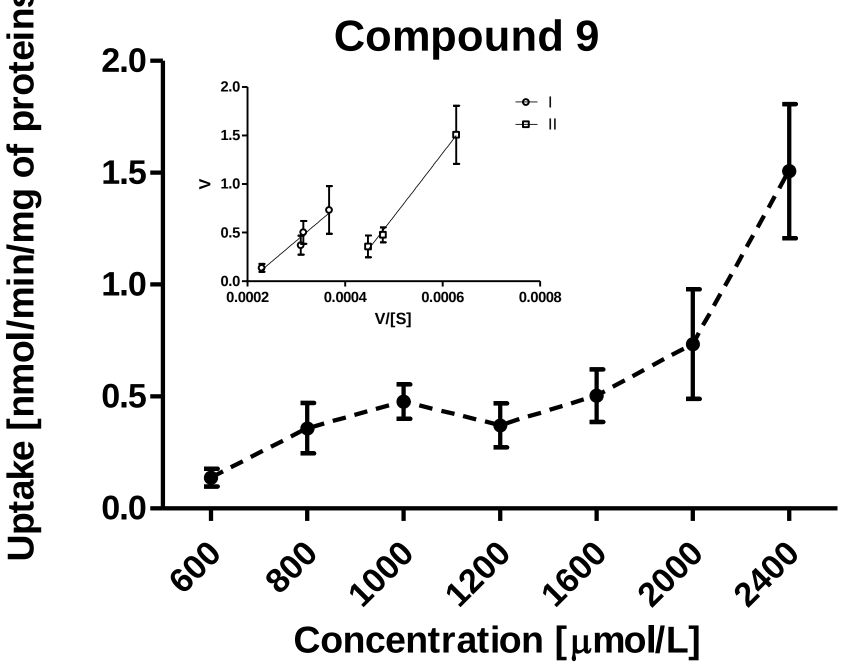

Figure S3. The uptake of selected sulfonamides into MCF-7 or MDA-MB-231 cells.

A) The uptake of compound 8 into MCF-7 cells and Eadie–Hofstee plots for OCTs mediated transport.

B) The uptake of compound 8 into MDA-MB-231 cells and Eadie–Hofstee plots for OCTs mediated transport.

C) The uptake of compound 9 into MCF-7 cells and Eadie–Hofstee plots.

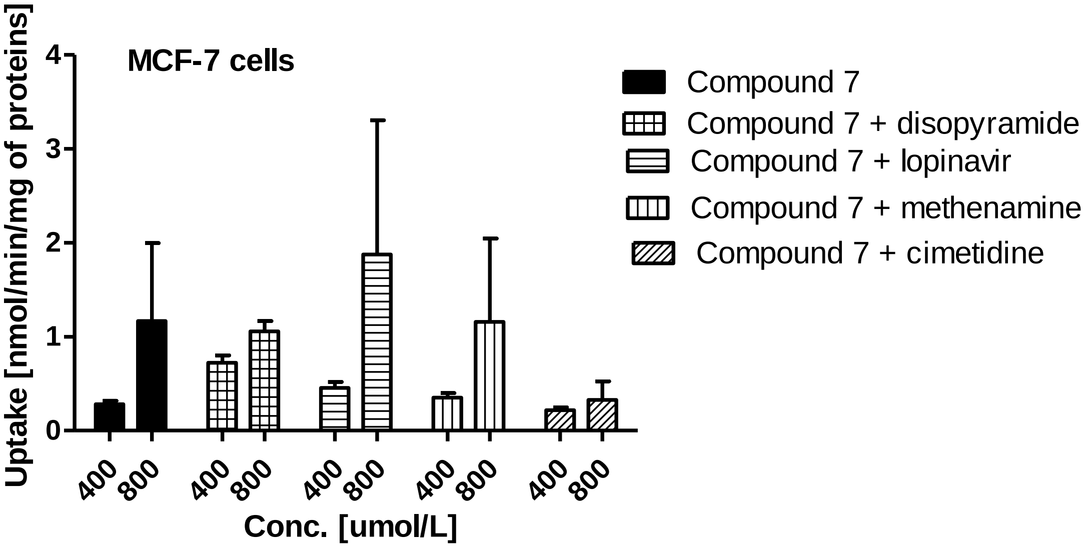
MCF-7 cells
Uptake [nmol/min/mg of proteins]
4
3
2
1
0
400
800
Conc. [umol/L]
Compound 7
Compound 7 + disopyramide
Compound 7 + lopinavir
Compound 7 + methenamine
Compound 7 + cimetidine

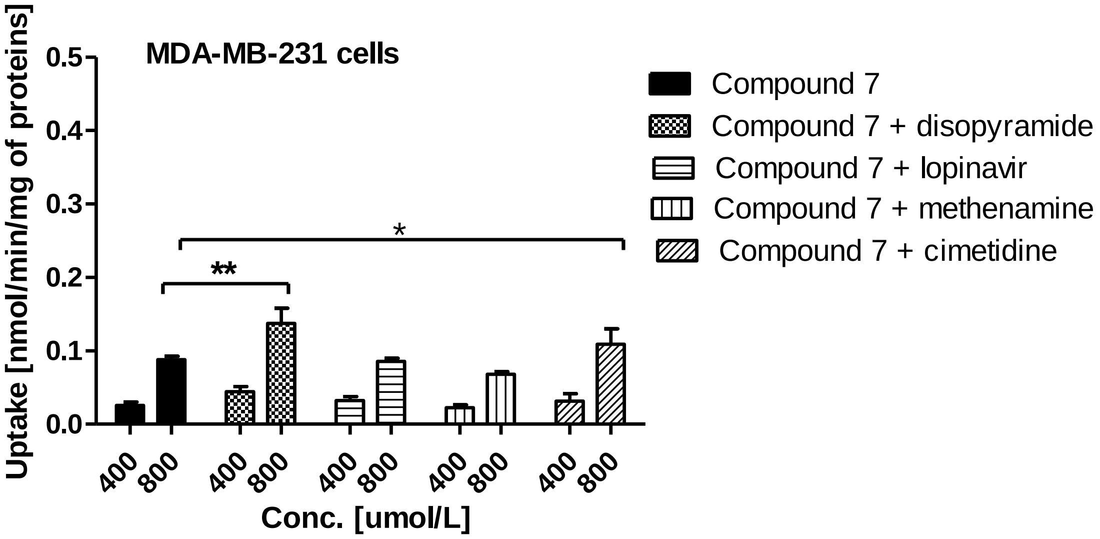
MDA-MB-231 cells
Uptake [nmol/min/mg of proteins]
0.5
0.4
0.3
0.2
0.1
0.0
*
**
400
800
Conc. [umol/L]
Compound 7
Compound 7 + disopyramide
Compound 7 + lopinavir
Compound 7 + methenamine
Compound 7 + cimetidine

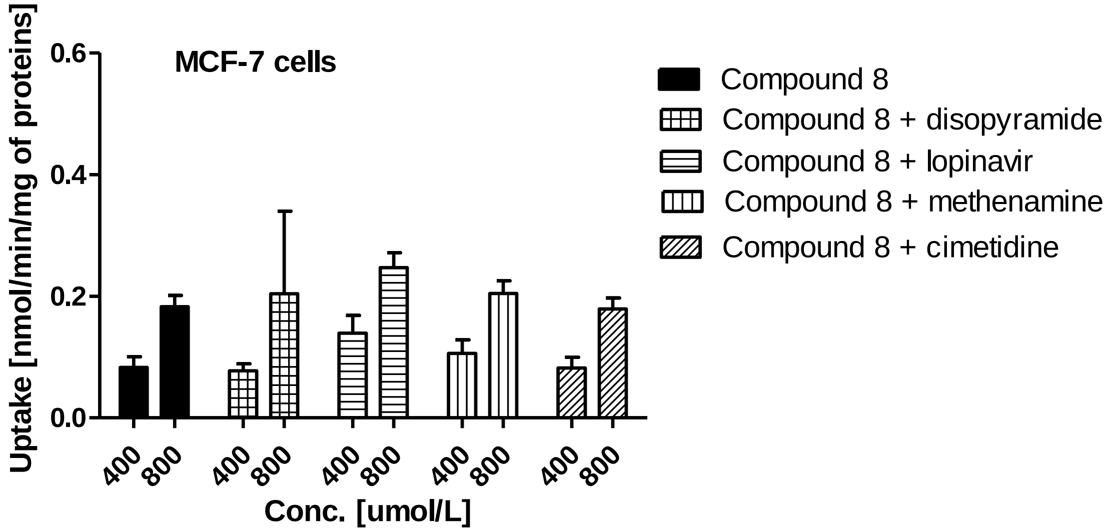
MCF-7 cells
Uptake [nmol/min/mg of proteins]
0.6
0.4
0.2
0.0
400
800
Conc. [umol/L]
Compound 8
Compound 8 + disopyramide
Compound 8 + lopinavir
Compound 8 + methenamine
Compound 8 + cimetidine

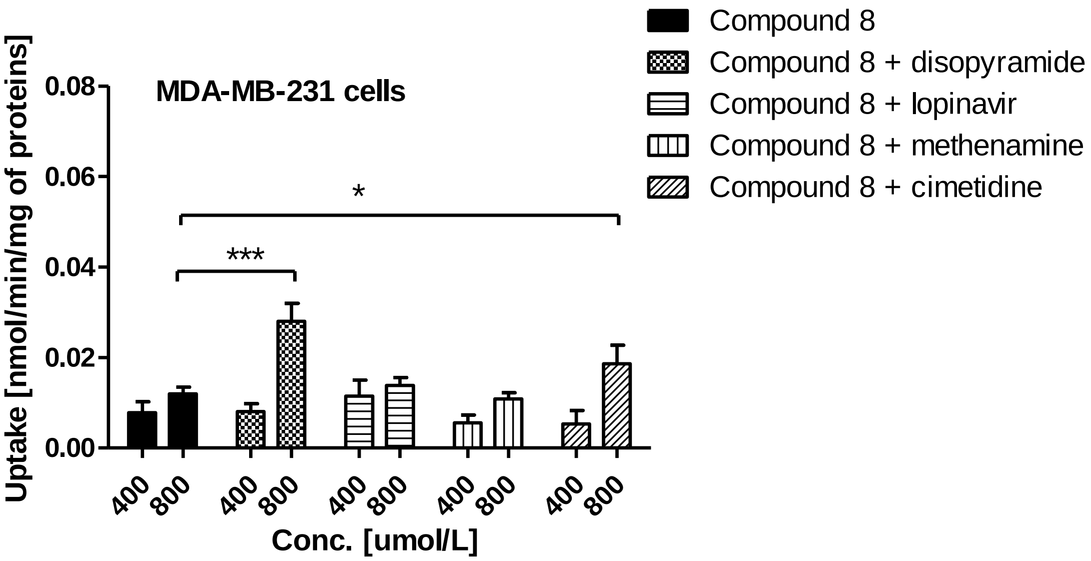
MDA-MB-231 cells
Uptake [nmol/min/mg of proteins]
0.08
0.06
0.04
0.02
0.00
*
***
400
800
Conc. [umol/L]
Compound 8
Compound 8 + disopyramide
Compound 8 + lopinavir
Compound 8 + methenamine
Compound 8 + cimetidine

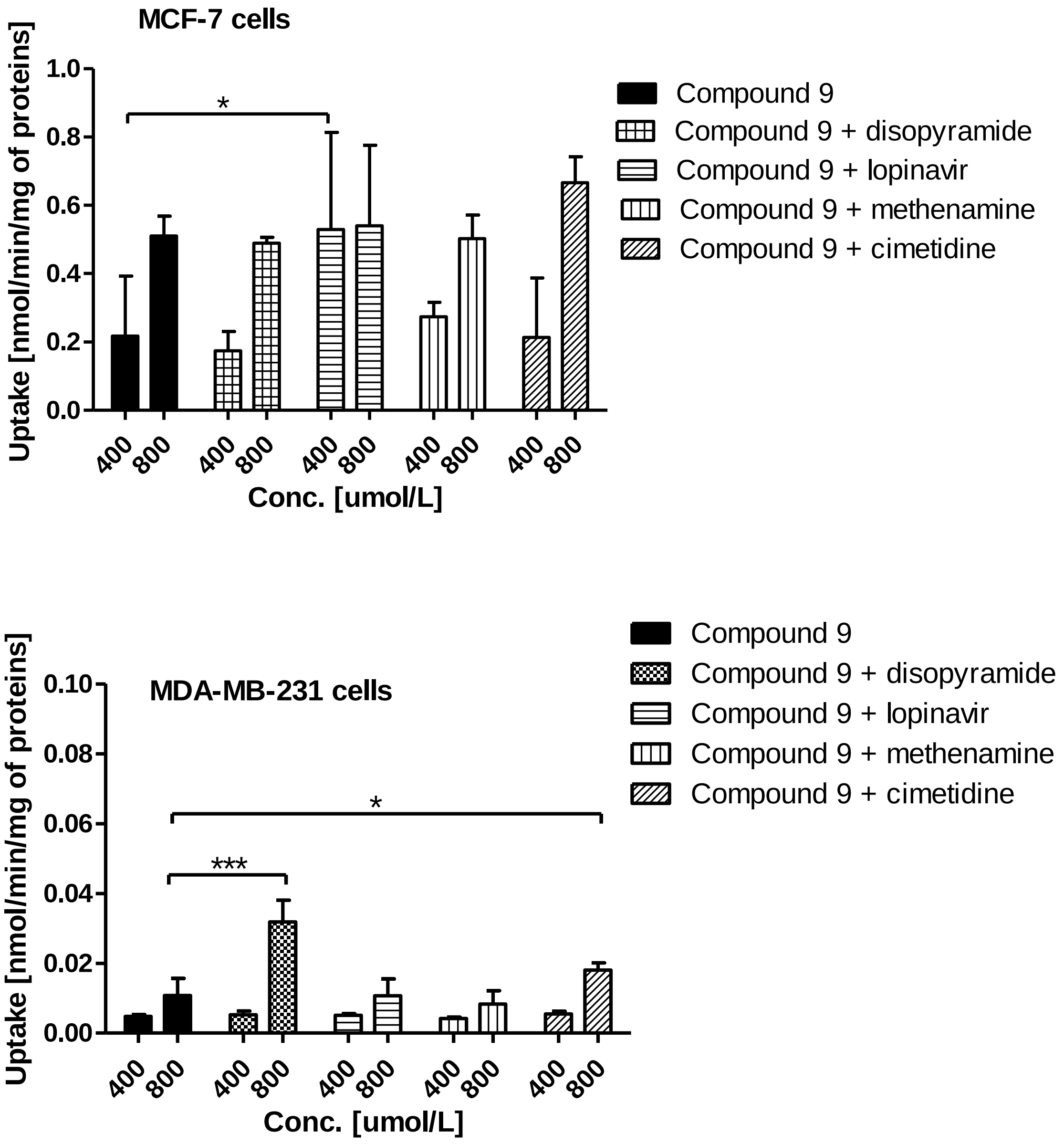

Figure S4. The uptake mechanism of compounds **7** - **9** (400 and 800 µmol/L) into MCF-7 cells and MDA-MB-231 cells. The uptake was determined in the presence of OCT and MATE inhibitors, disopyramide, lopinavir, methenamine, cimetidine (400 and 800 µmol/L) for 10 minutes at 37 °C. One-way Anova analysis was performed to compare the uptake of pure compounds (**7**–**9** at 400 and 800 µmol/L) with their uptake in the presence of transporters inhibitors. The significant differences between the uptake of pure compounds **7**–**9** (black bars) and their respective mixtures with inhibitors (disopyramide, lopinavir, methenamine or cimetidine) are marked with black lines and are denoted with asterisk. * $p < 0.05$, ** $p < 0.01$, *** $p < 0.001$.
